# Direct Image Feature Extraction and Multivariate Analysis for Crystallization Process Characterization

**DOI:** 10.1021/acs.cgd.1c01118

**Published:** 2022-03-19

**Authors:** Frederik J. S. Doerr, Cameron J. Brown, Alastair J. Florence

**Affiliations:** † Technology and Innovation Centre, EPSRC CMAC Future Manufacturing Research Hub, 99 George Street, Glasgow G1 1RD, U.K.; ‡ Strathclyde Institute of Pharmacy & Biomedical Sciences, University of Strathclyde, Glasgow G4 0RE, U.K.

## Abstract

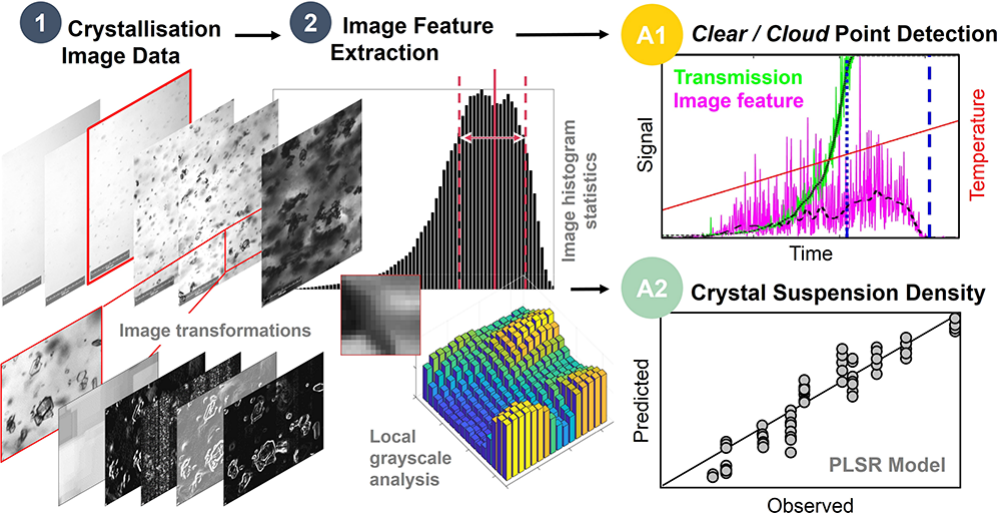

Small-scale crystallization experiments (1–8 mL) are widely used during early-stage crystallization process development to obtain initial information on solubility, metastable zone width, as well as attainable nucleation and/or growth kinetics in a material-efficient manner. Digital imaging is used to monitor these experiments either providing qualitative information or for object detection coupled with size and shape characterization. In this study, a novel approach for the routine characterization of image data from such crystallization experiments is presented employing methodologies for direct image feature extraction. A total of 80 image features were extracted based on simple image statistics, histogram parametrization, and a series of targeted image transformations to assess local grayscale characteristics. These features were utilized for applications of *clear*/*cloud* point detection and crystal suspension density prediction. Compared to commonly used transmission-based methods (mean absolute error 8.99 mg/mL), the image-based detection method is significantly more accurate for *clear* and *cloud* point detection with a mean absolute error of 0.42 mg/mL against a manually assessed ground truth. Extracted image features were further used as part of a partial least-squares regression (PLSR) model to successfully predict crystal suspension densities up to 40 mg/mL (*R*^2^ > 0.81, *Q*^2^ > 0.83). These quantitative measurements reliably provide crucial information on composition and kinetics for early parameter estimation and process modeling. The image analysis methodologies have a great potential to be translated to other imaging techniques for process monitoring of key physical parameters to accelerate the development and control of particle/crystallization processes.

## Abstract 2

A novel methodology for crystallization image data analysis using direct image features and multivariate analysis for accurate *clear*/*cloud* point detection and crystal suspension density prediction is reported. Direct image features are based on global image statistics, histogram parametrization, and a series of targeted image transformations to assess local grayscale characteristics.

## 1 Introduction

Crystallization is a purification and separation process widely applied in the agricultural, pharmaceutical, and chemical industries during the production of specialty or high-value chemicals.^1^ The successful development, optimization, and control of crystallization processes rely on the accurate determination of solid–liquid phase equilibria, system thermodynamics, rate process kinetics, and solid-state chemistry. Process design decisions can have a direct impact on the robustness and stability of the crystallization process,^2^ production yields,^2^ purity,^3^ as well as downstream isolation^4^ and performance attributes of the crystalline material, which could influence the overall therapeutic efficacy as part of the formulated final product.^5^

Process analytical technology (PAT) is increasingly applied for the analysis, monitoring, and control of pharmaceutical manufacturing processes with guidance from regulatory agencies encouraging the integration of PAT during process implementation.^6^ In the context of crystallization, strategies for the quantification of crystal suspension densities are mostly derived from solute concentration measurements using spectroscopic techniques such as UV/vis, Raman, or infrared.^7,8^ Focused beam reflectance measurements (FBRM) can be used to monitor particle size distribution (PSD) trajectories *in situ* avoiding the need for sampling, sample transfer, and offline measurements. However, the chord length distribution data from FBRM needs to be transformed to access the PSD^9^ and is therefore often only used semiquantitatively to assess relative changes in the particle counts across highly discretized size fractions. More recently, techniques including optical imaging and FBRM have been used to successfully quantify particle size and suspension densities up to 10 wt %;^10^ however, this approach combines measurements from multiple PAT probes, which is not feasible for small-scale experiments.

Optical imaging itself is a popular PAT application to monitor manufacturing processes involving solid–liquid, mixed phase systems such as particle suspensions during crystallization. Combined with automated image processing and analysis methodologies, data from optical imaging can be exploited to extract quantitative information on relevant particle properties related to size and shape.^11−14^ Various commercial PAT systems are available for process imaging with probes directly submerged into the process stream to enable imaging of suspended particles. These off-the-shelf commercial systems typically include proprietary image processing and analysis software for size and shape quantification. Image analysis methods to detect individual crystals/particles during image processing and to extract size and shape information on suspended particles are commonly limited in their applicability to low suspension densities where the edges of individual particles can still be easily detected, and the effects of extensive particle–particle overlapping in the images remain negligible.^15^ Object detection also commonly relies on image processing methods for noise reduction, e.g., local smoothing using image filters or despeckling after binarization, which can significantly impact the quantification accuracy of sensitive particle descriptors^16^ and which reduce the accuracy of these image analysis methods to detect early nucleation where only a few small crystalline particles might be present inside the image focal plane. There are examples using simple image statistics as a basis for process monitoring and quantification without the need for extensive image processing. In these cases, local concentrations or suspension densities are directly correlated to individual, extracted image pixel intensity statistics, most commonly the mean gray level intensity.^17−20^ This approach however has not yet been further expanded to include a wider range of optimized image features which might improve its ability to resolve physical changes in the system.

Small-scale experiments (1–8 mL) are often used during early-stage process development to obtain basic information on solute solubility, metastable zone width, as well as first estimates on nucleation and growth kinetics.^2^ The availability of commercial, automated, and parallel reactor systems allows such experiments to be carried out at the milliliter scale in a material sparing manner. However, the small dimensions of the vessels limit the use of high-end PAT equipment designed for applications in larger-scale crystallizers. Instead, transmission measurements are commonly used to monitor each experiment and detect *clear* and *cloud* points related to full solid dissolution or initial nucleation/precipitation, respectively.^2,21,22^

Given the value of accurate data describing the thermodynamics and kinetics of crystal formation at small scale, an analysis framework based on image features has been developed which can be employed to complement traditional object detection methods and enhance the information content accessible through standard optical imaging techniques. A total of 80 image features are part of this data-driven computer vision approach and were extracted from each image using targeted image transformations, parametrization of the pixel intensity histogram, and basic image intensity statistics. These methodologies were utilized to analyze a series of image data sets from small-scale experiments of mefenamic acid in solvent mixtures of diglyme and water (70:30–90:10, w/w). The image analysis framework was applied (A1) to support the accurate detection of early nucleation (*cloud* point) and complete dissolution at the solubility line (*clear* point) as well as (A2) to provide estimates of crystal suspension densities using extracted image features as input variables for a partial least-squares regression (PLSR) model. The work further includes an experimental design proposal for in-sample PLSR calibration using a stepwise heating protocol.

## 2 Materials and Experimental Methods

### 2.1 Materials

Mefenamic acid (MFA) was sourced from Sigma (Merck KGaA, Darmstadt, Germany, Lot#MKCH3607). Suspensions with changing MFA weight ratios were prepared in a 5 g mixture of diglyme (DIG, bis(2-methoxyethyl) ether) and deionized water (WAT) with ratios of 70:30, w/w (DIG70), 80:20, w/w (DIG80) or 90:10, w/w (DIG90). DIG was sourced from Fisher Scientific (ACROS Organics, Waltham, United States). WAT was purified and deionized using a Milli-Q system (Merck KGaA, Germany). Details of all prepared samples for this study are tabulated in Table 1.

**Table 1:** MFA Samples Prepared with a Mixture of DIG and WAT with Solvent Ratios between 70:30, w/w and 90:10, w/w

experiment	*x*_DIG_ [wt %]	*x*_WAT_ [wt %]	*m*_MFA_ [mg]	*m*_Solv_[g]	*x*_MFA_ [g/g]
MFA-DT	70	30	262.4	5	0.052
MFA-70-1	70	30	262.4	5	0.052
MFA-70-2	70	30	190.5	5	0.038
MFA-70-3	70	30	133.7	5	0.027
MFA-70-4	70	30	70.3	5	0.014
MFA-80-1	80	20	391.3	5	0.078
MFA-80-2	80	20	301.3	5	0.060
MFA-80-3	80	20	221.0	5	0.044
MFA-90-1	90	10	748.5	5	0.150
MFA-90-2	90	10	601.5	5	0.120
MFA-90-3	90	10	502.9	5	0.101
MFA-90-4	90	10	421.2	5	0.084
MFA-90-5	90	10	363.5	5	0.073

### 2.2 Crystalline

Small-scale crystallization experiments were conducted in 8 mL clear glass vials (height 61 mm × diameter 16.6 mm, hydrolytic class 1) inserted in a Crystalline (Technobis Crystallization Systems, Alkmaar, The Netherlands). Mixing was provided through magnetic stirring with a bottom stirring speed of 700 rpm using a PTFE-coated elliptical stirrer (dimensions 10 × 3 mm). In general, fast and homogeneous mixing of most crystal suspensions can be assumed for these small-scale experiments. The Crystalline platform itself was equipped with a temperature controller, a transmissivity sensor, and an imaging system (RR-PV module). Reactor jacket temperature and transmission measurements were recorded at 1 Hz. A tuning step was applied to the transmission signal when the solution reaches clear point (i.e., crystals are fully dissolved). This essentially sets the laser power to 100% transmission. The transmission signal was further processed to reduce noise using a Hampel filter with a moving window size (*k*_Hpl_) of 7 and an outlier criterion of *n*_Hpl,σ_ = 3 local standard deviations as well as a moving average filter (Savitzky-Golay, order 1) with a moving window size (*k*_SG_) of 9. For the processed transmission signal, *clear* and *cloud* points were detected at a threshold of >99.9% transmissivity_sm_ and <99.9% transmissivity_sm_, respectively. The imaging frequency was user-defined with frame-rates between 0.5 and 0.05 fps depending on the expected process dynamics during crystallization. At the end of each experiment, all process data and images with a resolution of 480 × 640 px at an image pixel size of 2.8 μm/px were exported for offline data processing.

**Figure 1:**
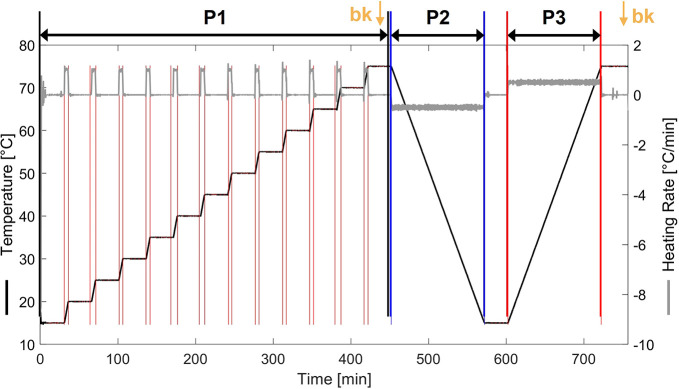
Crystalline experiments with programmed temperature profile. (P1) Step-wise heating sequence (heating 1 K/min) with 30 min isothermal conditions for in-sample PLSR calibration. (P2) Metastable zone width experiment with crystal nucleation detection (slow cooling −0.5 K/min) and (P3) dissolution profile for solubility temperature detection (slow heating at 0.5 K/min). bk (annotated with yellow arrow) shows where background image data were collected at the end of P1 and P3.

Temperature profiles for the small-scale experiments were designed for image feature calibration and to characterize basic crystallization (thermo)dynamics during cooling and heating. An example of the implemented Crystalline temperature profile for each vessel is shown in Figure 1 with additional details for each experiment provided in the Supporting Information (Table S1). Each of the three phases of the applied profile, P1–3 is described further. (P1) Each experiment was designed to undergo an initial stepwise heating sequence with heating steps of 5 K and isothermal data collection at holding periods of 30 min. Images for PLSR calibration were collected at the end of each isothermal temperature stage in P1 after an equilibration time of 5 min. The calibrated PLSR model was used to predict crystal suspension densities during dynamic crystallization events. A constant cooling/heating rate of ±0.5 K was used for (P2) metastable zone width assessment and (P3) solubility determination. For MFA-DT, isothermal holding periods were extended to 120 min to create an image data set for initial method development.

### 2.3 Image Processing and Analysis

All routines for automated image processing and analysis were implemented in MATLAB (R2019b, Mathworks). Multicore, parallel processing of individual 2D images was used to accelerate routine image analysis. Image feature extraction focuses on the quantification of image characteristics from eight-bit grayscale image data collected during each experimental run. A total of 80 image features are extracted using three distinct approaches: (1) directly from the image raw data based on global image intensity statistics (*n* = 6), (2) through a parametrization of the pixel intensity histogram (*n* = 15), and (3) from a variety of targeted image transformations or local image variance analyses (*n* = 59).^23−34^ Image transformations provide an excellent opportunity to explore a diversity of image features for target applications, e.g., by adjusting kernel parameters which were optimized in the context of crystal suspension characterization. An overview of all 80 extracted image features with additional details on the employed parameters is provided in Table S2 (Supporting Information). Fifteen background images of each experiment from clear solution were averaged and used to define a divergence criterion for *clear* and *cloud* point detection. These background images were automatically selected at the highest temperature under conditions of complete dissolution (denoted by points bk in Figure 1). To assess image feature applications for *clear* and *cloud* point detection, a user-defined ground truth was assessed manually from the collected crystallization image data.

### 2.4 Partial Least Square Regression (PLSR)

Partial least square regression (PLSR) analysis was employed for crystal suspension density prediction using the extracted image features. The crystal suspension density (*x*_c_) is defined here as the weight of suspended crystalline solids per volume element of the solvent liquid phase due to the nature of the data acquisition approach imaging a fixed suspension volume but with changing solvent compositions (DIG70–DIG90). Effects of solute molecules in the liquid phase and the contribution of crystalline material to the overall suspension density are assumed to be equal across all solvent systems.

An implementation of the SIMPLS algorithm was used for PLSR which calculates the PLSR factors directly as linear combinations of the original variables^35^ and therefore retains good interpretability for future efforts on feature selection and/or expansion. For PLSR, image feature data from isothermal conditions during an initial stepwise heating procedure were isolated (Figure 1 P1). Discrete binning and averaging were used to assess prediction improvements by reducing image-to-image variability. A bin size of *k*_av_ = 5 images was selected to compensate for potential image-to-image variability while retaining good time resolution during dynamic crystallization events. The image feature data were mean-centered prior to PLSR model training and testing. The number of latent variables (ncomp) was optimized based on the calculated mean square error of the prediction during cross-validation (MSECV). The PLSR model performance to accurately predict crystal suspension densities was assessed against (I) potential image-to-image variability and (II) data set-to-data set variability. (I) Feature data from individual images of a single image data set, MFA-DT, were randomly divided into training and test data (85:15). The training data were used to optimize the number of latent variables (ncomp) during 10-fold cross-validation. (II) Individual image data sets (MFA-70-1–MFA-90-5) were randomly divided into training and test data sets (10:2). The training data sets were used to optimize the number of latent variables (ncomp) during four-fold cross-validation.

## 3 Results and Discussion

### 3.1 Image Feature Extraction and Statistics

**Figure 2:**
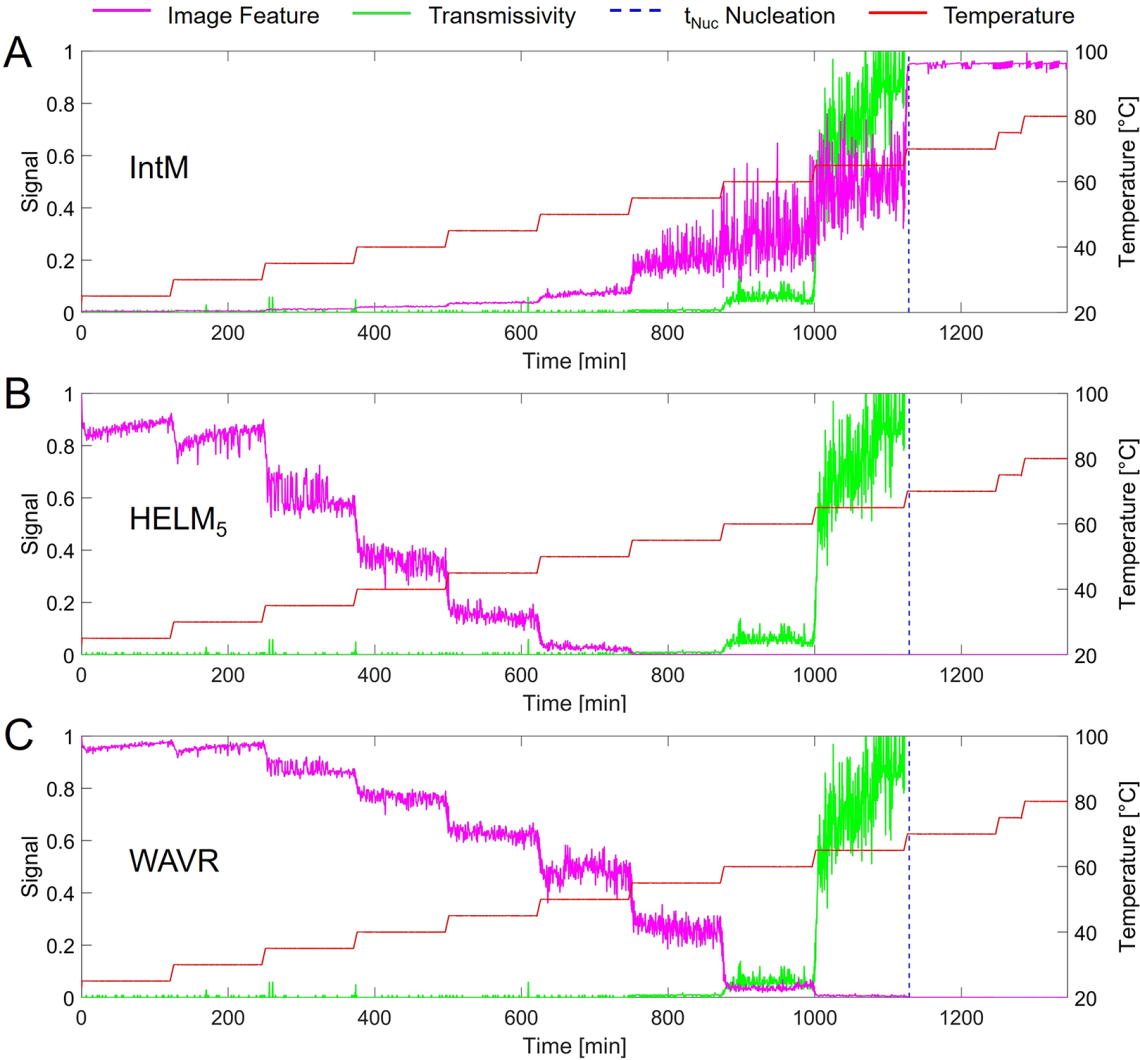
Time-resolved (green) transmission signal compared to selected (magenta) image feature signals during stepwise heating (red line, temperature): (A) mean image intensity (IntM), (B) HELM_5_, and (C) WAVR. Manually identified *clear* point (blue dashed, complete dissolution).

Image features were extracted using 31 methods aiming to quantify image properties from basic image intensity statistics (*n* = 6), from a parametrization of the pixel intensity histogram (*n* = 15) and from image transformations operating within a local gray level pixel neighborhood (*n* = 59). Features extracted from targeted image transformations, e.g., using Prewitt^23^ or Sobel^24^ operators were of particular interest. These image transformations are often employed for edge detection in computer vision applications and therefore might be highly sensitive to physical changes in the crystal suspension. Many of the methods tested in this study provide multiple image features or metrics which might include mean pixel intensity, variance, or entropy from each of the transformed images. In total, 80 image features were extracted and assessed. Three selected feature signals are visualized in Figure 2 to illustrate the complexity of the data analysis problem but also opportunities for correlating image features with physical changes in the sample during a stepwise heating profile. These include (A) the mean image intensity of the raw data (IntM) often used during image analysis as a basic image feature, (B) HELM_5_ assessing pixel intensity variance against the local mean background used for *clear* and *cloud* point detection, and (C) WAVR from a 2-D wavelet decomposition with high peak resolution across changing suspension densities. Each feature signal exhibits unique characteristics related to their temporal signal intensity and variance.

**Figure 3:**
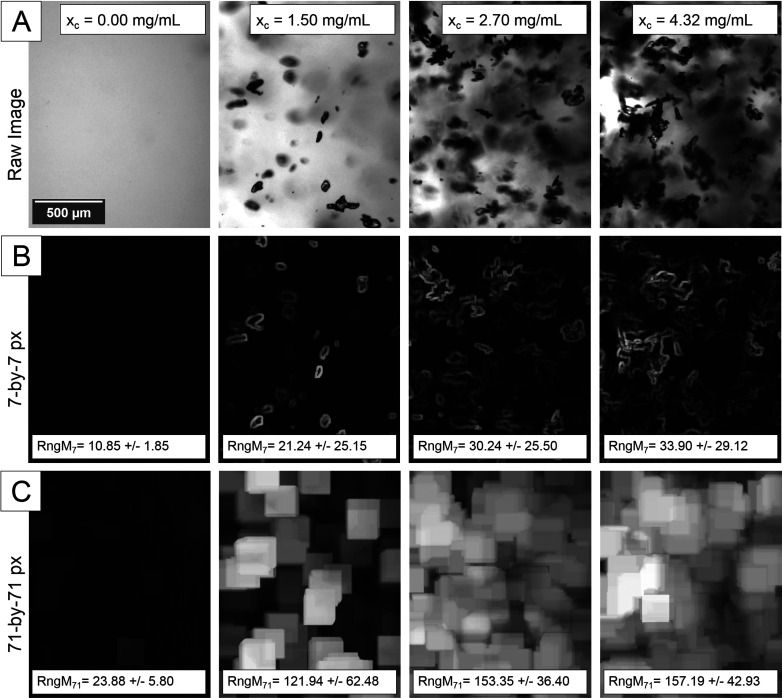
(A) Raw images of crystal suspensions with changing MFA suspension density between *x*_c_ = 0.00 and 4.32 mg/mL. Image transformation using range filters with a changing kernel neighborhood size of (B) 7 × 7 px and (C) 71 × 71 px. The transformed image with a small filter kernel resembles the raw image with reversed polarity highlighting local pixel intensity gradients (RngM = mean pixel intensity). In contrast, a range filter with larger kernels is more sensitive to the presence of crystals. Different kernel sizes can be used to tailor image feature sensitivity for changing crystal suspension densities.

Most image transformations employ application-specific kernels which operate within a defined pixel neighborhood. The kernel shape, size, and values can be optimized to capture image attributes of interest, e.g., for texture or pattern recognition. For crystal suspensions, for example, Figure 3 visualizes the impact of changing kernel sizes for a range filter with a kernel size of (Figure 3B, Rng_7_) 7 × 7 px and (Figure 3C, Rng_71_) 71 × 71 px, respectively. While a smaller kernel size of 7 × 7 px can be used for edge enhancement to get an indication of the number of crystalline objects, larger kernel sizes of 71 × 71 px are very sensitive to local intensity fluctuations in the presence of only a few suspended crystalline objects. Therefore, this filter seems particularly useful for the characterization of images from crystallization experiments with medium and dilute crystal suspension densities and for early detection of particle formation, respectively. Other image filters follow similar methodologies and can be tailored using user-defined parameters which were partially preoptimized based on collected crystallization images in the MFA-DT data set. An overview of all methods for image feature extraction and additional details on the image filter kernel parameters are provided in Table S2 (Supporting Information).

The performance of each extracted image feature to resolve physical changes during crystallization was assessed and compared through a calculation of the peak resolution (Res) between pairs of collected measurement distributions with known, discrete changes in the crystal suspension density. Res was calculated as shown in eq 1 assuming normal distribution:
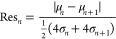
1where μ and σ are the mean and the standard deviation of the image feature distributions, respectively, for samples *n* and *n* + 1. When Res is equal to 1, 95% of the measured feature values can be correctly classified between the two samples. Therefore, it is desirable to maintain a Res value greater than 1 across all changes in crystal suspension density.

**Figure 4:**
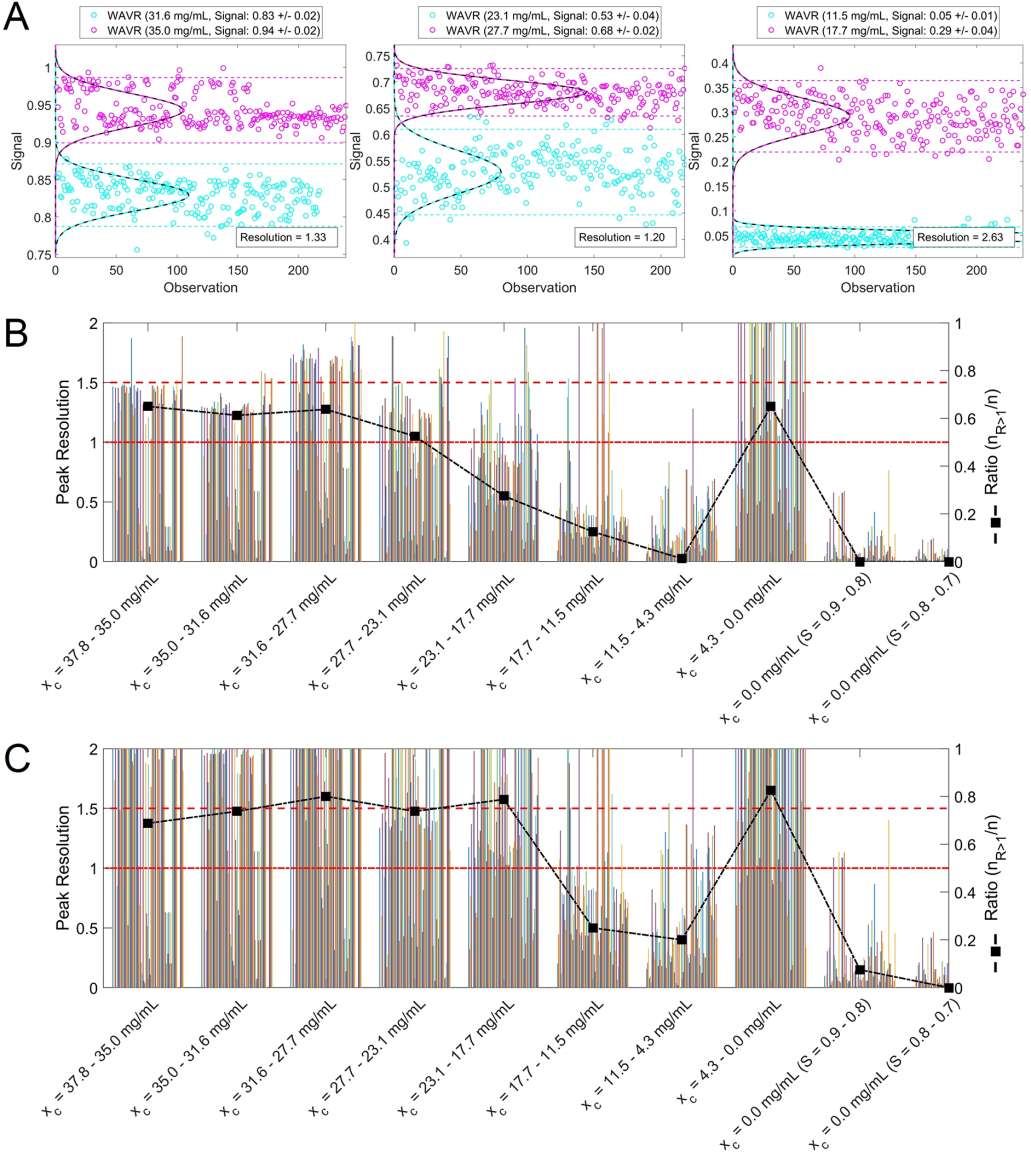
Pair-wise assessment of peak resolution for all image feature data distributions to distinguish between discrete suspension densities (*x*_c_) between 37.8 and 0.0 mg/mL (*S* = 0.7). (A) Three examples of WAVR comparing data distribution metrics and peak resolution. Dashed line indicates measured signal distribution at μ ± 2σ. Peak resolution of all features are shown in (B) for feature data extracted from raw images and (C) after using a discrete binning and image averaging (*k*_av_ = 5) for noise reduction. Black squares indicate the ratio of image features with a peak resolution higher than 1 (*n*_R>1_/*n*).

Image feature resolution was assessed using the MFA-DT image data set with 11 distinct isothermal temperature levels between 30 °C (*x*_c_ = 37.8 mg/mL) and 80 °C (*x*_c_ = 0.0 mg/mL, *S* = 0.7) during a stepwise heating protocol (example shown in Figure 1 P1). Expected crystal suspension densities were calculated based on the MFA phase diagram presented in Figure 6. During each isothermal period, an average of 239 images were collected. Figure 4A shows example measurement distributions with pairwise resolution assessment for WAVR. The results indicate that between each crystal suspension density pair (magenta versus cyan) the distributions of WAVR values are sufficiently different to distinguish between them, which is reflected in a Res value greater than 1 for all cases. Figure 4B expands upon this concept and shows the resolution values across all features for the raw image data and in Figure 4C the resolution values across all features after using discretized binning to reduce image feature noise (*k*_av_ = 5). The black trend line indicates the ratio of image features that achieve a Res > 1.

In general, the ability to distinguish changes in the crystal suspension density based on image features in MFA-DT is not uniform but strongly depends on the crystal suspension density itself. Interestingly, images with very high suspension density (*x*_c_ > 23.1 mg/mL) show the highest resolution between the collected measurement distributions and maintain a Res >1, Figure 4B. At suspension densities between 23.1 and 4.3 mg/mL, the peak resolution indicates that the distribution of image feature values is insufficiently separated to distinguish between discrete changes in the crystal suspension density. This is likely due to having few particles in suspension, resulting in a reduced probability of particles appearing in these images and an increased influence related to the size and shape of each individual particle itself. Feature resolution in this range with more dilute suspensions can be improved using discretized binning, Figure 4C. Differences between *x*_c_ = 4.3 mg/mL and *x*_c_ = 0.0 mg/mL (*S* = 0.9, no particles), i.e., comparing image feature signals above and below the MFA solubility, show a good peak resolution at this crucial point during dissolution (*clear* point) with 65% of all image features exhibiting a resolution of Res > 1.

A ranking of all image features with numeric values from the resolution assessment is provided in Table S3 and Table S4 (Supporting Information) for the raw data and after discretized binning for noise reduction (*k*_av_ = 5), respectively. Each table further includes the determined Pearson correlation coefficient (PCC) to quantify the overall linear correlation between each image feature and the calculated crystal suspension density as the response variable. Among them, WAVR (rank 1) exhibits a high positive linear correlation (PCC = 0.99) with a consistent peak resolution of Res > 1 across the full range of suspension densities between *x*_c_ = 37.8 mg/mL and *x*_c_ = 0.0 mg/mL (*S* = 0.9). Notably, a number of image features outperform the basic mean image intensity (IntM, rank 19, Res(*k*_av_ = 5) = 2.16 ± 1.10 ∈ [0.61, 4.28], PCC = −0.94) and the transmission signal (not ranked, Res(*k*_av_ = 5) = 0.93 ± 1.09 ∈ [0.01, 2.86], PCC = −0.69) in terms of their ability to resolve physical changes in the crystal suspension density especially for high suspension densities up to 37.80 mg/mL, confirming the value of advanced image parametrization and targeted feature engineering efforts. Example images for each supersaturation level under isothermal conditions are shown in Figure S1 and Figure S2 (Supporting Information), respectively. The histogram for the images in Figure S2 was adjusted (stretched) to visualize subtle differences in the image intensities for the reader, which are quantified during image feature extraction but can otherwise not be directly observed in the raw images.

The performance of individual image features might be impacted through the optical properties of the crystalline material in this study. Additionally, differences in the particle size distribution and the particle shape could affect image feature performance where size and shape features are significantly larger than the image pixel size. Therefore, the performance of individual image features as part of this data-driven approach for crystallization image analysis needs to be re-evaluated for new compound systems. Depending on the desired application, features with low resolution and low correlation to real physical changes in the system can be excluded to accelerate image data analysis for a routine or real-time implementation. Compared to traditional object detection methods to determine number density, size, and shape information, extracted image features are not inherently meaningful. The following two applications aim to further explore the use of extracted image features for (A1) detection of complete dissolution (*clear point*) or crystal nucleation (*cloud* point) and (A2) crystal suspension density prediction.

### 3.2 Application (A1) - *clear* and *cloud* Point Detection

Accurate *clear* and *cloud* point detection is crucial to obtain information on the compound solubility and metastable zone width which are often used as the basis for process development and optimization. Small-scale crystallization experiments typically rely on transmission measurements to automatically detect complete crystal dissolution (*clear* point) or initial crystal nucleation (*cloud* point) using a user-defined, fixed threshold level. This first application explores the use of extracted image features as an alternative method to transmission to further improve *clear* and *cloud* point detection accuracy.

A rational method for feature selection is essential to identify robust image feature(s) for *clear* and *cloud* point detection. A simple single feature threshold might be used as a detection criterion to facilitate rapid, routine analysis. The median background value of each image feature (*m*_*y*_*i*_,*bk*_) was calculated using clear background images of the solution. Consequently, *cloud* points were detected once the feature signal diverges from *m*_*y*_*i*_,*bk*_ at a threshold related to the background signal’s assessed standard deviation (*y*_*i*_ > |*m*_*y*_*i*_,*bk*_ ± *n*_Nuc_·σ_*y*_*i*_,*bk*_|). For *clear* point detection, the inverse objective, i.e. a convergence criterion, was used instead.

Feature signal processing can help to increase the signal-to-noise ratio (SNR) of extracted image features and, therefore, improve the overall accuracy and precision of detection. Two filters were selected for preprocessing the extracted raw signals of all 80 image features: (1) a Hampel filter for local outlier detection^36^ caused by individual images with strong image feature fluctuations, e.g., related to foreign particles or insoluble impurities in the suspension and (2) a moving average filter for local smoothing of random noise (Savitzky-Golay, order 1). Three filter parameters, the optimum window size of each moving filter (*k*_Hpl_, *k*_SG_), and the outlier criterion for the Hampel filter (*n*_Hpl,σ_ = number of local standard deviations) were subsequently optimized against a user-defined ground truth for three randomly selected image data sets (MFA-70-3, MFA-80-2, and MFA-90-4) with the objective to minimize the temperature mean squared error (MSE) for *clear* and *cloud* point detection. For the detection criterion, the divergence/convergence threshold was defined at three different levels with *n*_Nuc_ of 4, 8, and 16 to further evaluate the overall robustness of this approach.

**Figure 5:**
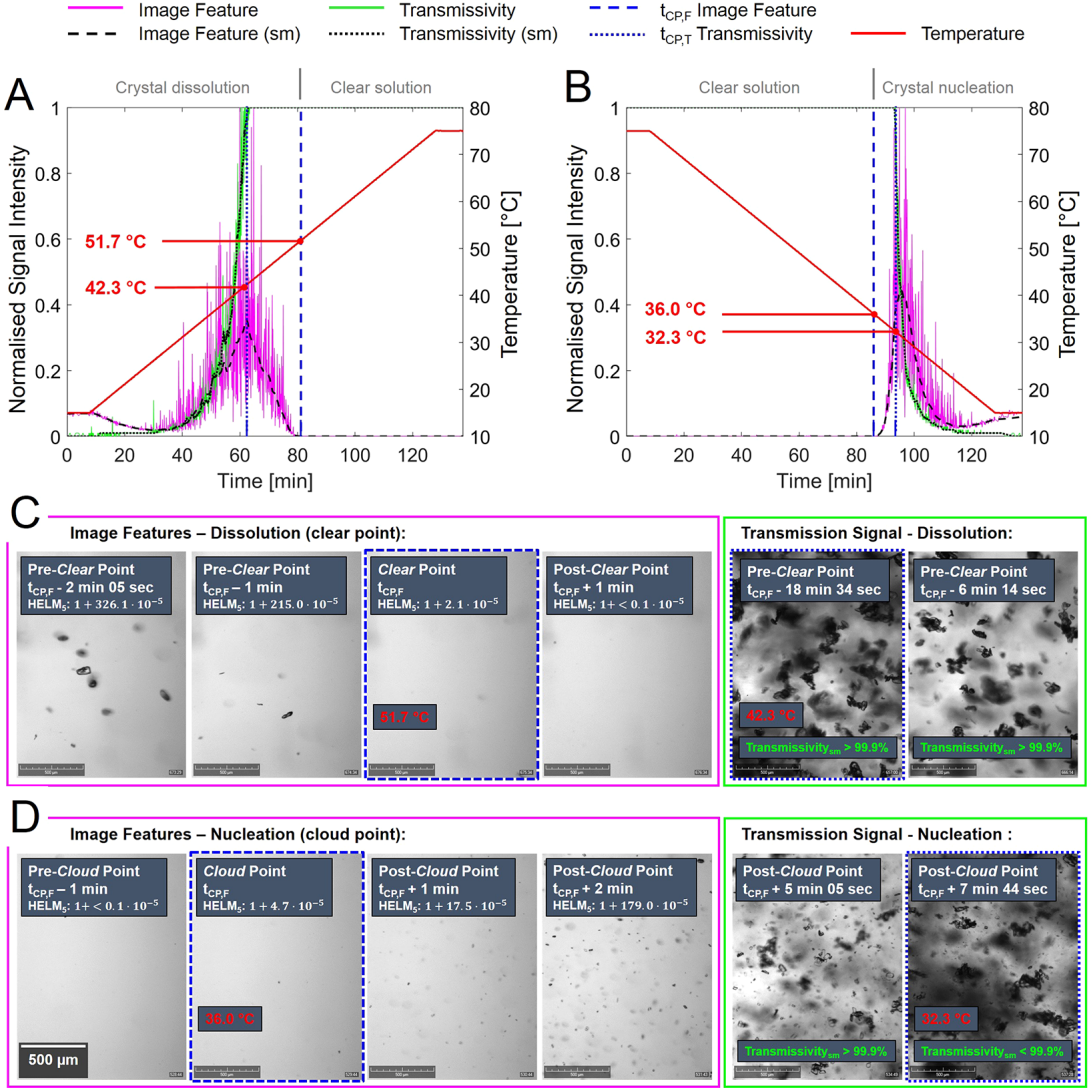
Comparison of (A) *clear* point (complete dissolution) and (B) *cloud* point (nucleation) detection from (green) a transmission-based signal and (magenta) HELM_5_ image feature signal at a heating/cooling rate of 0.5 K/min. (Black, sm) After signal processing. (Blue) Detected *clear* and *cloud* point for each method (*t*_CP_). Selected images of the sample’s image time-series undergoing (C) complete dissolution (*clear* point detection) and (D) nucleation (*cloud* point detection) are shown for visual comparison and verification.

HELM_5_ was identified as the best performing image feature for *clear* and *cloud* point detection with an overall MSE of 0.05 °C^2^ after preprocessing the image feature data with *k*_Hpl_ = 29, *n*_Hpl,σ_ = 0.1, and *k*_SG_ = 11. Details about the performance of all features for *clear* and *cloud* point detection and feature-specific optimized signal preprocessing parameters (*k*_Hpl_, *n*_Hpl,σ_, and *k*_SG_) are provided in Table S5 (Supporting Information). Among them, BREN (Brenner’s focus) and PRWV (Prewitt operator) also show excellent performance. In contrast to HELM_5_, which aims to quantify absolute differences related to its background intensity, BREN and PRWV are based on an assessment of the local spatial intensity gradient where they are traditionally used to assess image focus or for edge detection, respectively. Low performing image features are related to global image intensity statistics such as parameters from the image intensity histogram (HistSpan, HistSumBK) or the image intensity index of dispersion (IntIdxD). These image features do not operate within defined image pixel neighborhoods and, therefore, are less likely to distinguish random global image intensity fluctuations from highly localized intensity fluctuations due to individual crystals in the imaging field of view. Figure 5 shows the application of HELM_5_ for MFA-80-2 during (A) slow heating at 0.5 K/min and (B) slow cooling at −0.5 K/min in direct comparison to a transmission-based *clear* and *cloud* point detection method. The processed, smoothed feature signal (black dashed line) shows significantly reduced local fluctuations compared to the feature raw signal (magenta line); however, the strong performance of HELM_5_ for *clear* and *cloud* point detection is related to its consistent background signal of 1.00 ± 6.71 × 10^–5^ (*S* = 0.7–0.9). The consistent background signal of HELM_5_ is related to the nature of this image feature which quantifies the average ratio between pixel intensities and the background mean gray level intensity of its pixel neighborhood (for HELM_5_ a 5 × 5 pixel kernel), therefore, compensating the impact of local random noise and small intensity fluctuations. Image series in Figure 5C,D are provided to give an impression of the dissolution and nucleation dynamics. For the transmission signal (green box), the detected *clear* point with a threshold of >99.9% and *cloud* point with a threshold of <99.9% (blue dotted picture frames) after signal processing (transmissivity_sm_) are shown alongside images from intermediate time points to visualize the progress of crystal dissolution and crystal nucleation/growth, respectively. The image-based detection method shows an excellent performance with a detected *clear* point at 51.7 °C (complete dissolution, solubility) and a *cloud* point at 36.0 °C (initial nucleation, metastable zone width) in stark contrast to the transmission signal with a significantly reduced sensitivity to detect low crystal suspensions densities providing highly misleading *clear*/*cloud* point values.

**Figure 6:**
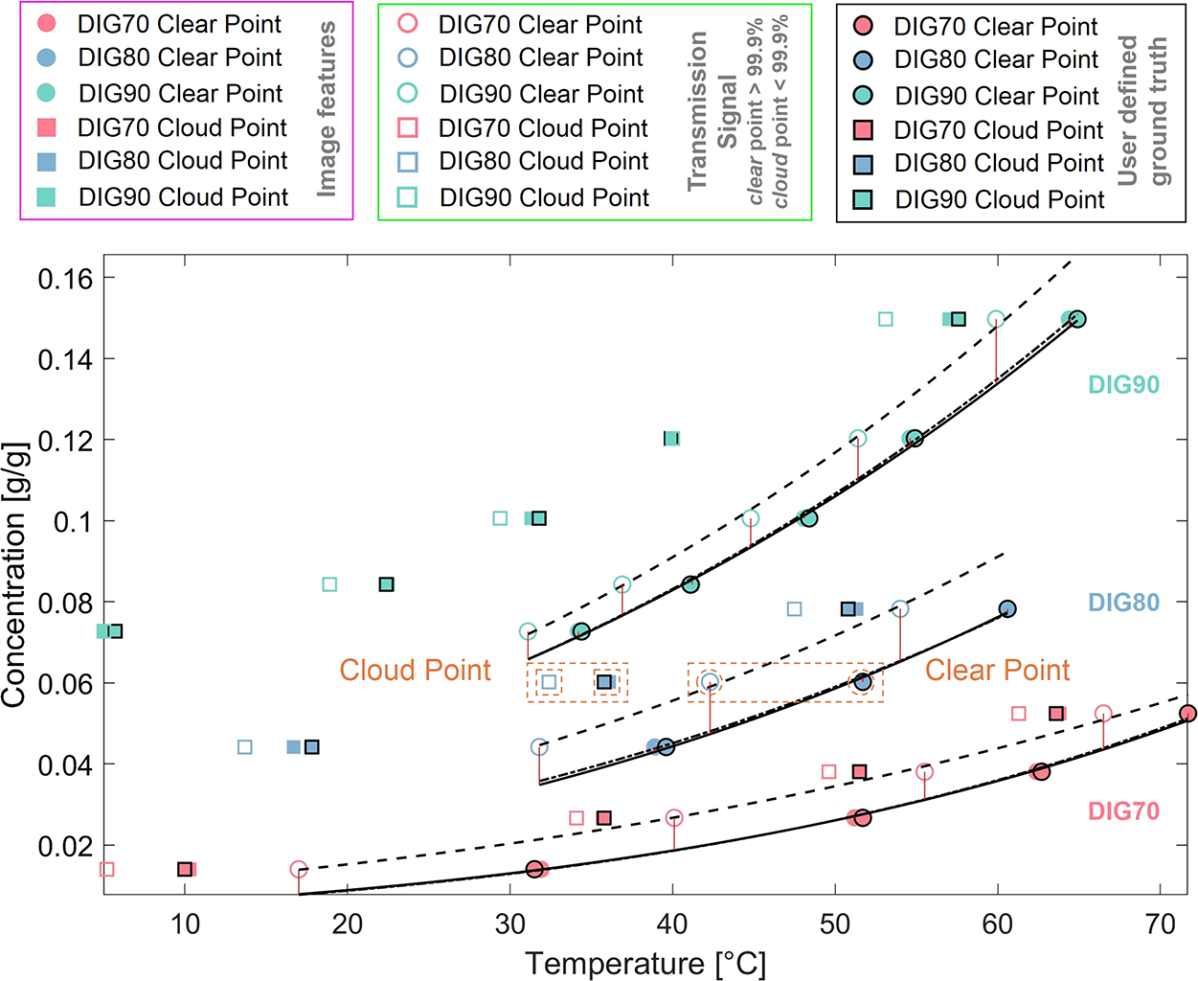
Phase diagram showing the detected *clear* and *cloud* points using (filled markers) image features, (empty markers) transmission signal and (filled markers with black edge) the user-defined ground truth. The examples marked in orange for dissolution (*clear* point) and nucleation detection (*cloud* point) are presented in detail in Figure 5. Van’t Hoff solubility prediction from (dash-dotted line) image feature-based *clear* points, (dashed line) transmission-based *clear* points and (solid line) the user-defined ground truth. (Red lines) Absolute detection error.

Figure 6 further shows the impact of both *clear* and *cloud* point detection methods on the phase diagram of MFA constructed from a series of 12 small-scale experiments (MFA-70-1–MFA-90-5). Image feature-based *clear* and *cloud* points (Figure 6 filled symbols) are consistently shifted to higher temperatures, indicating later detection of complete dissolution during heating and earlier initial nucleation detection during cooling compared to a detection based on the transmission signal with a 99.9% threshold (Figure 6 empty symbols). Across all data sets (MFA-70-1–MFA-90-5), the average difference between both methods, HELM_5_ image feature versus transmission signal, is 6.63 ± 3.61 °C ∈ [3.10 °C, 14.90 °C] and 2.59 ± 1.56 °C ∈ [0.00 °C, 5.10 °C] for *clear* and *cloud* point detection, respectively. Image feature-based *clear* and *cloud* points are consistent against a manually assessed, user-defined ground truth (filled markers with black edge).

The van’t Hoff equation for nonideal solutions (eq 2) was used to interpret the experimental solubility data (*clear* points) where *x* is the mole fraction of the solute in the solution, *T* is the temperature, *R* is the ideal gas constant, *ΔH*_d_ is the enthalpy of dissolution, and *ΔS*_d_ is the entropy of dissolution.^1^
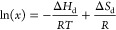
2

Point estimates for *ΔH*_d_ and *ΔS*_d_ from a regression analysis of each individual solvent composition were used to generate solubility curves in Figure 6 for (dashed line) transmission-based *clear* points, (dash-dotted line) image feature-based *clear* points and (solid line) the user-defined ground truth. Additional details of the regression analysis are provided in Figure S3 (Supporting Information).

In the context of the van’t Hoff solubility model, one can distinguish and quantify the contribution of systematic and random measurement errors related to the overall accuracy and precision of the detection method. The results indicate a good fit of eq 2 to the experimental data with comparatively low unexplained, random error contributions for each of the applied detection methods (*R*_adj_^2^ ≥ 0.99, residual mean squared error (RMSE) ≤ 3.82 × 10^–2^). The systematic error (bias) of each detection method was assessed using the estimated solubility model parameters *ΔH*_d_ and *ΔS*_d_ (user-defined ground truth *ΔH*_d_ ∈ [20.05 kJ/mol, 28.14 kJ/mol], *ΔS*_d_ ∈ [34.09 J/(mol K),42.92 J/(mol K)]), which are both increasing with higher solvent ratios of water, consistent with other studies investigating MFA solubility in water and a range of organic solvents.^37^ Across all solvent compositions, the method for image feature-based solubility detection has an error of −0.08 ± 0.68 kJ/mol and −0.20 ± 2.09 J/(mol K) for *ΔH*_d_ and *ΔS*_d_, respectively. In contrast, the transmission-based solubility detection method has a significantly higher systematic error of −2.90 ± 3.59 kJ/mol and −7.38 ± 10.38 J/(mol K) for *ΔH*_d_ and *ΔS*_d_, respectively.

On the basis of the experiments performed in this study (*y*_*i*_, *n* = 12) and using the predicted solubility of the established MFA phase diagram (*ŷ*_gTruth_(*T*_*i*_)), the observed mean absolute detection error can be expressed as the mean crystal suspension density at the detected *clear* point for each detection method (MAE = *y*_*i*_ – *ŷ*_gTruth_, Figure 6 red lines). The image feature-based approach has an MAE of <0.001 ± 0.001 g/g ∈ [−0.001, 0.002] or 0.42 ± 1.16 mg/mL ∈ [−1.13, 2.00] and, therefore, aligns well with the manually determined *clear* points (ground truth). For the transmission-based *clear* points detected at a 99.9% transmission threshold, the MAE is 0.009 ± 0.003 g/g ∈ [0.006,0.016] or 8.99 ± 2.92 mg/mL ∈ [5.86, 15.26], indicating an incorrect, early detection during constant heating at 0.5 K/min with an extensive crystal mass still present in the sample. This confirms initial qualitative observations presented in Figure 5C (green box) showing significant amounts of crystalline particles at the detected *clear* point using the transmission-based method. Similar observations with an improved determination of the clear point using image-based detection have been described in the literature after collected image data sets from small-scale crystallization experiments were manually reviewed and compared.^38,39^

Incorrect *clear* and *cloud* point detection significantly affects the determined compound solubility or metastable zone width and therefore crystallization process design decisions through error propagation further emphasizing the importance of accurate and precise metrics for *clear* and *cloud* point detection. The results have demonstrated that image features such as HELM_5_ can be used as an alternative to transmission-based detection methods and provide a more reliable determination of these basic (thermo)dynamic properties for new compounds during early-stage, small-scale crystallization process development.

### 3.3 Application (A2) - Suspension Density Prediction

Crystal suspension density measurements are useful for crystallization model development to inform system dynamics during parameter estimation or can be used for direct crystallization process monitoring and control. However, a quantification of the crystal suspension density is not readily accessible via direct measurement during small-scale, highly parallelized experiments. This second application aims to explore the use of image features for the prediction of crystal suspension densities from crystallization image data. Crystal suspension densities are defined here as the weight of suspended crystalline solids per volume of the solvent liquid phase (*x*_c_ in mg/mL) based on the nature of the data acquisition approach imaging a fixed suspension volume but with changing solvent compositions (DIG70–DIG90).

PLSR analysis is a multivariate analysis method which utilizes latent variables to model response (target) variables from changes in the input (explanatory) variables. Two PLSR models were trained and tested using image features as input variables: (I) training and test data were randomly selected from the MFA-DT image data set to assess the impact of image-to-image variability on the PLSR prediction accuracy. (II) Training and test data consist of randomly selected independent data sets (training MFA-70-1 to MFA-90-5 excluding test data MFA-80-3 and MFA-90-5) and was used to explore the ability to translate a pretrained PLSR model to new crystallization experiments, therefore assessing the impact of variability between different, independent image data sets on the PLSR prediction accuracy. The MFA phase diagram in Figure 6 was used to calculate expected crystal suspension densities under isothermal conditions during stepwise heating for all image data sets (see example temperature profile Figure 1 P1). Image feature preprocessing was limited to discrete binning and averaging and subsequent mean-centering.

**Figure 7:**
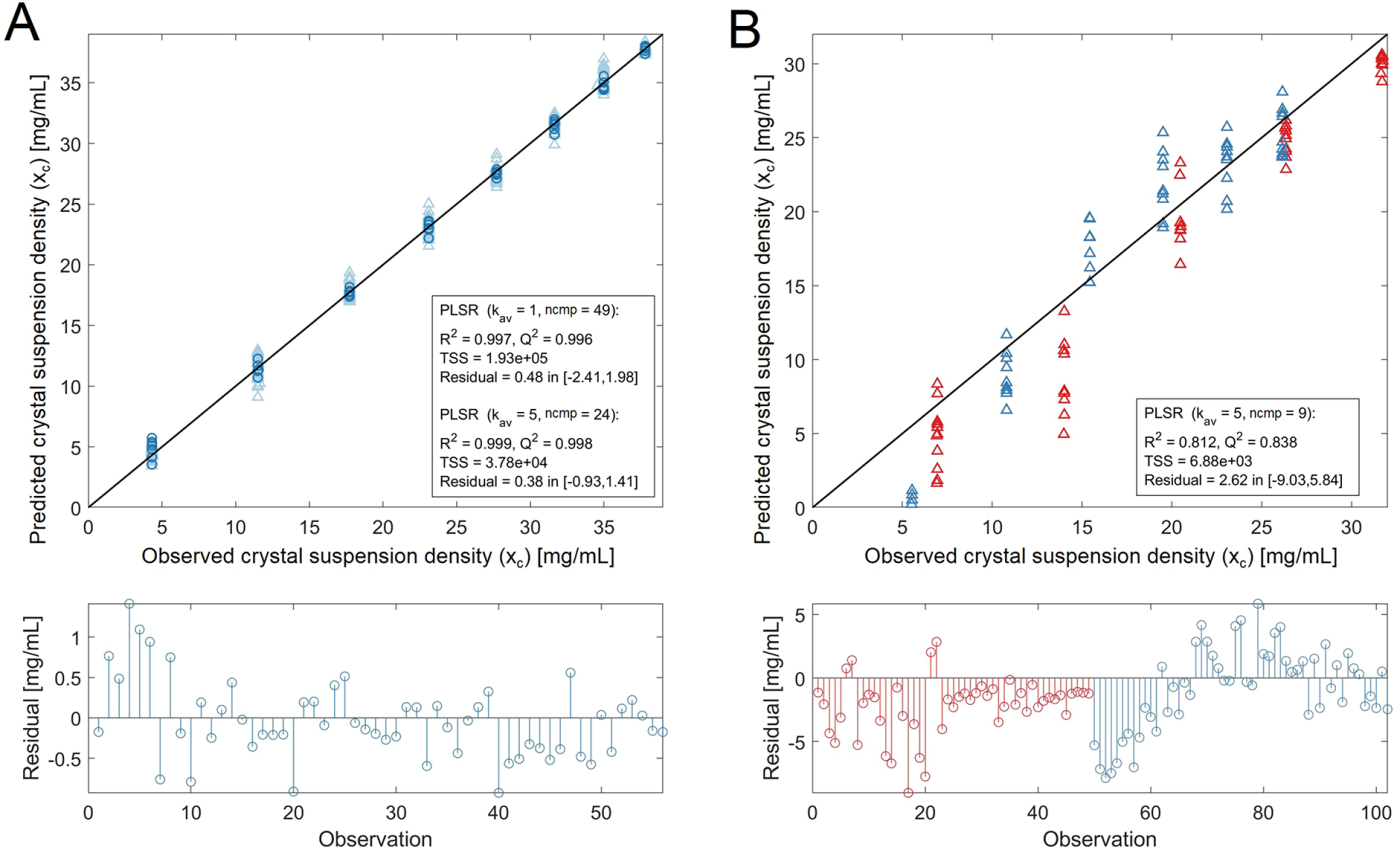
(Top) Parity plots and (bottom) residuals from crystal suspension density predictions using image features within a PLSR model. (A) Tested with *n*_Img_ = 280 (light blue, *k*_av_ = 1) and *n*_Img_ = 56 (dark blue, *k*_av_ = 5) randomized observations (= images). Train and test data set are taken from the same stepwise heating sequence. (B) Test data from two independent experiments, (red) MFA-80-3 and (blue) MFA-90-5 (*n*_Img_ = 92, *k*_av_ = 5).

Figure 7 shows (top) parity plots and (bottom) residuals for both PLSR models. Using binned image features (*k*_av_ = 5), it is possible to predict crystal suspension densities with a mean residual error of 0.38 and 2.62 mg/mL assessed using test data (I, Figure 7A) from the same data set and (II, Figure 7B) from fully independent experiments, respectively. Image-to-image variability does not seem to have a significant impact on the PLSR prediction comparing performance metrics in Figure 7A using (light blue) no binning and (dark blue) a bin size of *k*_av_ = 5. In contrast, residuals of independent data sets (Figure 7B bottom, red and blue) show a nonrandom error distribution, indicating that some parts of the variability in the response cannot be accurately predicted using the pretrained PLSR model. This nonrandom noise could be attributed to small differences in the crystal suspension which are not fully captured such as its specific particle size and shape distribution which however have an impact on extracted image features. The number of latent variables is significantly reduced using a PLSR training data set with image feature data from multiple, independent data sets as shown in Figure 7B (ncmp = 9), indicating a lower risk for overfitting. Absolute PLSR weights (R) are highest for TENG, LAPV, RngGaussV_157_, IntV, and GLLV_5_ (for details, see Figure S4). TENG (Tenengrad function^33^) is a gradient magnitude-based method involving the Sobel operator. LAPV is the calculated image variance after the Laplacian operator is applied to determine second derivatives for passing high spatial frequencies associated with sharp edges. All other image features aim to quantify local (RngGaussV, GLLV) and global (IntV) image intensity variance. Interestingly, while the absolute gradient magnitude seems to be well captured using TENG, multiple image features are used to provide a good correlation between observed changes in the suspension density and the local or global image intensity variance. Differences in the quantification of the image intensity variance between the individual image features are mainly related to the type and the size of the convolution filter used during image transformation, which consequently both could be used as targets for future, application-specific feature engineering efforts. In the case of RngGaussV, a range filter is used during the image transformation. The impact of changing kernel sizes for range filters was discussed in the context of crystallization image characterization with example images provided in Figure 3. Overall, the pretrained PLSR model in Figure 7B is able to explain most physical changes in both independent data test sets (*goodness of prediction*, *Q*^2^ = 0.838), and the relative prediction error is most significant for highly diluted crystal suspensions. While out of the scope for this study, additional information from object detection and characterization methods could be used to potentially improve crystal suspension density predictions for these diluted crystal suspensions.

Besides size and shape information, crystal suspension density predictions provide details on the system’s material balance to help inform population balance models during parameter estimation. Our results have shown for the first time that these can be derived from crystallization image data, significantly improving the scope of image data analysis applications for process characterization.

## 4 Conclusions

An image analysis framework for the routine characterization of crystallization image data was developed to extract a total of 80 image features from targeted image transformations including a parametrization of the pixel intensity histogram and other basic image intensity statistics. The developed methodologies were used to analyze images collected during small-scale crystallization experiments of MFA in changing solvent mixtures of DIG and WAT (70:30–90:10, w/w). Extracted image features were assessed for two important applications, A1 and A2, representative of key stages in crystallization process development. Specifically, the features were used for (A1) *clear* and *cloud* point detection to determine the MFA phase diagram and (A2) as input variables for a PLSR model to estimate crystal suspension densities up to 40 mg/mL.

(A1) For *clear* (*n* = 12) and *cloud* point (*n* = 12) detection, a simple threshold for the image feature signal HELM_5_ was able to reliably detect full dissolution and onsets of crystal nucleation with a mean absolute error of 0.42 °C compared to a manually assessed, user-defined ground truth. For image feature-based *clear* point detection, the mean absolute error was even smaller with 0.19 °C or 0.42 g/mL. The image feature-based detection method significantly improves the determination of solubility and metastable zone width compared to the often employed transmission-based detection method which was unable to detect crystal suspension densities up to 15.26 mg/mL with a mean absolute error of 5.80 °C compared to the user-defined ground truth.

(A2) The use of image features for PLSR model prediction of crystal suspension densities aims to provide additional crucial information during crystallization. Image-to-image variability had a low impact on the PLSR model performance which was assessed using training and test data from a single image data set and which can be further improved reducing random noise through discretized binning (*k*_av_ = 5, *Q*^2^ = 0.998). The ability to predict crystal suspension densities using a pretrained PLSR model was further tested using two fully independent image feature data sets (*k*_av_ = 5). The pretrained PLSR model is able to successfully predict physical changes in both independent data test sets using image features; however, prediction accuracy is reduced (*Q*^2^ = 0.838) with nonrandom error distributions.

The implemented image analysis framework provides capabilities to automatically and accurately interrogate routinely collected image data from crystallization experiments. It is therefore significantly expanding the use of image analysis methodologies to complement traditionally employed methods focused on object detection. Such automated data analysis tools maximizing the useful information yield from small scale material sparing experiments have a significant role in enabling digital design approaches and the application of crystallization process modeling in early process development. Future efforts might focus on further exploring and developing targeted routines for image feature extraction of crystal suspension images to improve the accuracy and precision of pretrained PLSR models to independent crystallization image data sets. The image analysis framework also has great potential to be translated to other image-based systems and techniques for crystallization process characterization. Real-time image feature analysis could be applied for process control as a decision tool, reducing the need for more expensive, dedicated probes.

## Supporting Information Available

Direct image feature extraction and multivariate analysis for crystallization process characterization (PDF)

## Notes

Data underpinning this publication are openly available from the University of Strathclyde KnowledgeBase at https://doi.org/10.15129/75002e1c-80dc-42ab-984a-3444fbef9c03
