# Supplementary material for: Direct Image Feature Extraction and Multivariate Analysis for Crystallization Process Characterization
Source: Cryst Growth Des. 2022 Mar 19;22(4):2105–16. doi: 10.1021/acs.cgd.1c01118 (PMC8990522; doi:10.1021/acs.cgd.1c01118)
Supplement: Supplementary file 1 — cg1c01118_si_001.pdf [file cg1c01118_si_001.pdf]

Supporting Information for "Direct image feature extraction and multivariate analysis for crystallisation process characterisation"

Frederik J. S. Doerr[a,b], Cameron J. Brown[a,b], Alastair J. Florence[a,b,]

[a] *EPSRC CMAC Future Manufacturing Research Hub, Technology and Innovation Centre, 99 George Street, Glasgow, G1 1RD, UK.*
[b] *Strathclyde Institute of Pharmacy & Biomedical Sciences (SIPBS), University of Strathclyde, Glasgow, G4 0RE, UK.*

Contents

S1.1 Crystalline Experiments - Temperature profile S2
S1.2 Image features overview S4
S1.3 Image feature resolution S11
S1.4 Application (A1) - nucleation detection S18
S1.5 Application (A2) - suspension density prediction S25

Email address: alastair.florence@strath.ac.uk (Alastair J. Florence)
URL: www.cmac.ac.uk (Alastair J. Florence)

S1.1. Crystalline Experiments - Temperature profile

Table S1: Crystalline temperature profiles for small-scale crystallisation experiments. Stepwise heating with 5 K increments.

**ID**	**Temperature profile**
MFA-DT	Stepwise heating: 25°C → 75 °C at 1 K/min, 120 min isothermal,<br>Stepwise heating: 75°C → 80 °C at 1 K/min, 30 min isothermal
MFA-70-1	Stepwise heating: 25°C → 85 °C at 1 K/min, 30 min isothermal,<br>Constant cooling: 85°C → 25°C at -0.5 K/min,<br>Constant heating: 25°C → 85°C at 0.5 K/min
MFA-70-2	Stepwise heating: 15°C → 75 °C at 1 K/min, 30 min isothermal,<br>Constant cooling: 75°C → 15°C at -0.5 K/min,<br>Constant heating: 15°C → 75°C at 0.5 K/min
MFA-70-3	Stepwise heating: 5°C → 65 °C at 1 K/min, 30 min isothermal,<br>Constant cooling: 65°C → 5°C at -0.5 K/min,<br>Constant heating: 5°C → 65°C at 0.5 K/min
MFA-70-4	Stepwise heating: 5°C → 65 °C at 1 K/min, 30 min isothermal,<br>Constant cooling: 65°C → 5°C at -0.5 K/min,<br>Constant heating: 5°C → 65°C at 0.5 K/min
MFA-80-1	Stepwise heating: 15°C → 75 °C at 1 K/min, 30 min isothermal,<br>Constant cooling: 75°C → 15°C at -0.5 K/min,<br>Constant heating: 15°C → 75°C at 0.5 K/min
MFA-80-2	Stepwise heating: 15°C → 75 °C at 1 K/min, 30 min isothermal,<br>Constant cooling: 75°C → 15°C at -0.5 K/min,<br>Constant heating: 15°C → 75°C at 0.5 K/min

MFA-80-3	Stepwise heating: 5°C → 65 °C at 1 K/min, 30 min isothermal,<br>Constant cooling: 65°C → 5°C at -0.5 K/min,<br>Constant heating: 5°C → 65°C at 0.5 K/min
MFA-90-1	Stepwise heating: 40°C → 95°C at 1 K/min, 30 min isothermal,<br>Constant cooling: 95°C → 40°C at -0.5 K/min,<br>Constant heating: 40°C → 95°C at 0.5 K/min
MFA-90-2	Stepwise heating: 40°C → 95°C at 1 K/min, 30 min isothermal,<br>Constant cooling: 95°C → 40°C at -0.5 K/min,<br>Constant heating: 40°C → 95°C at 0.5 K/min
MFA-90-3	Stepwise heating: 25°C → 85 °C at 1 K/min, 30 min isothermal,<br>Constant cooling: 85°C → 25°C at -0.5 K/min,<br>Constant heating: 25°C → 85°C at 0.5 K/min
MFA-90-4	Stepwise heating: 15°C → 75 °C at 1 K/min, 30 min isothermal,<br>Constant cooling: 75°C → 15°C at -0.5 K/min,<br>Constant heating: 15°C → 75°C at 0.5 K/min
MFA-90-5	Stepwise heating: 5°C → 65 °C at 1 K/min, 30 min isothermal,<br>Constant cooling: 65°C → 5°C at -0.5 K/min,<br>Constant heating: 5°C → 65°C at 0.5 K/min

S1.2. Image features overview

Table S2: Overview of all image features extracted from the Crystalline image data. 37 methods were used to extract a total of 80 image features quantifying local variations in grayscale, properties of the image histogram or related to image intensity statistics.[a] MATLAB Image Processing Toolbox, [b] literature/-Mathworks File Exchange. Expanded kernel sizes were scaled using bilinear interpolation.

**Feature Method**	**Description**
Brenner's focus[b] [1, 2]<br>BREN	Measurement based on the first difference between a pixel and its neighbour two points away.<br>$\mathrm{BREN} = \frac{1}{NM}\sum^{M}\sum^{N}\lvert I(i,j+2) - I(i,j)\rvert^2$
Local image entropy[a]<br>EntFiltM, EntFiltV	Transformation where each output pixel contains the entropy value of a user-defined pixel neighborhood (7-by-7, 71-by-71).<br>$\mathrm{EntFiltM} = \frac{1}{NM}\sum^{M}\sum^{N} I_{\mathrm{E}}$,<br>$\mathrm{EntFiltV} = \frac{1}{NM-1}\sum^{M}\sum^{N}\lvert I_{\mathrm{E}} - \bar{I_{\mathrm{E}}}\rvert^2$<br>$I_{\mathrm{E}}(m,n) = -\sum_{k}^{w_n w_m} p_k \log_2(p_k)$
Gaussian derivative [b] [3]<br>GDER	Measurement based on the first-order Gaussian derivatives $G_x(m,n,\sigma)$ and $G_y(m,n,\sigma)$ at scale σ.<br>$\mathrm{GDER}(\sigma) = \frac{1}{NM}\sum_{m}^{M}\sum_{n}^{N}\left[I(m,n) * G_x(m,n,\sigma)\right]^2 + \left[I(m,n) * G_y(m,n,\sigma)\right]^2$

Co-occurrence matrix[a]<br>GLCM	Measurement based on the gray-level co-occurrence matrix (GLCM) by calculating how often a pixel with the intensity value i occurs in a specific spatial relationship to a pixel with the value j. (offset: $\Delta m = 5$, $\Delta n = 5$). Derived properties: Contrast (intensity of pixel to its neighbor), Correlation (pixel to its neighbor), Energy (sum of squared elements) and Homogeneity (distribution of elements on GLCM diagonal).<br>$\mathrm{GLCM}(m,n) = \sum^{N}\sum^{M} \begin{cases} 1, & \text{if } I(m,n) = i \text{ and } I(x+\Delta x, y+\Delta y) = j \\ 0, & \text{otherwise} \end{cases}$
Graylevel local variance[b] [4]<br>GLLV	Measurement based on the local pixel gradient variance (I_{lv}, 5-by-5, 15-by-15).<br>$\mathrm{GLLV} = \frac{1}{NM}\sum_{m}^{M}\sum_{n}^{N}\left[I_{\mathrm{lv}}(m,n) - \bar{I_{\mathrm{lv}}}\right]^2$<br>$I_{\mathrm{lv}}(m,n) = \frac{1}{w_n w_m}\sum_{i}^{w_n}\sum_{j}^{w_m}\left[I(m+i, n+j) - \bar{I}\ \right]^2$
Graylevel variance[b] [5]<br>GLVA	Measurement assessing the global pixel intensity variance.<br>$\mathrm{GLVA} = \frac{1}{NM}\sum_{m}^{M}\sum_{n}^{N}\left[I(m,n) - \bar{I}\ \right]^2$
Energy of gradient[b] [6]<br>GRAE	Measurement based on the normalised pixel intensity gradient.<br>$\mathrm{GRAE} = \frac{1}{NM}\sum^{M}\sum^{N}\left(g_x^2 + g_y^2\right)$<br>$g_x(x,y) = g_i(x+1, y) - g_i(x,y)$<br>$g_y(x,y) = g_i(x, y+1) - g_i(x,y)$
Thresholded gradient[b] [2]<br>GRAT	Measurement based on the first difference and is summed for values larger than a certain threshold (ν).<br>$\mathrm{GRAT} = \frac{1}{NM}\sum^{M}\sum^{N}\lvert\nabla I(m,n)\rvert \text{ while } \lvert\nabla I(m,n)\rvert \geq \nu$

Squared gradient[b] [2]<br>GRAS	Measurement based on the squared line gradient (g_x).<br>$\text{GRAS} = \frac{1}{NM}\sum^{M}\sum^{N}\left(g_x^2\right)$<br>$g_x(x,y) = g_i(x+1,y) - g_i(x,y)$
Helmli's mean[b] [7]<br>HELM,<br>HELV	Measurement of mean local contrast by calculating the ratio between pixel intensities and the background mean gray level intensity of its pixel neighborhood (w_n and w_m, 5-by-5 pixel, 15-by-15 pixel).<br>$\text{HELM} = \frac{1}{NM}\sum^{M}\sum^{N} J$, $\text{HELV} = \frac{1}{NM-1}\sum^{M}\sum^{N} \lvert J - \bar{J}\rvert^2$<br>$J(m,n) = [I(m,n)/I_{\text{lm}}]$<br>$I_{\text{lm}}(m,n) = \frac{1}{w_n w_m}\sum_{i}^{w_n}\sum_{j}^{w_m} I(m+i, n+j)$
Histogram entropy[a] [5]<br>HISE	Measurement based on the global image pixel entropy (E).<br>$\text{E} = -\sum^{M}\sum^{N} p(I(m,n))\log_2(p(I(m,n)))$
Histogram range[b] [8]<br>HISR	Measurement based on the difference between the maximum and the minimum pixel intensity in the image (H_k = image histogram).<br>$\text{HISR} = \max(k \mid H_k > 0) - \min(k \mid H_k > 0)$

Histogram statistics<br>HistSumQ1-HistSumQ4,<br>HistSumBK,<br>Hist10-Hist90,<br>HistSpan	Measurement based on a parameterisation of the image intensity histogram (H_k). Histogram quartiles (in % of bin range): HistSumQ1 for $> 0\%$ to $< 25\%$, HistSumQ2 for $> 25\%$ to $< 50\%$, HistSumQ3 for $> 50\%$ to $< 75\%$ and HistSumQ4 for $> 75\%$ to $< 100\%$<br>HistSumBK $= \sum(k \vert H_k = 0)$<br>Histogram transformed to a cumulative distribution (Q0): Hist10 to Hist90 at Q0 = 0.1 to 0.9, HistSpan = (Hist90-Hist10)/Hist50
Intensity statistics[a]<br>IntMean, IntVar,<br>IntMedian, IntIdxD	Basic pixel intensity statistic assessing mean pixel intensity, pixel intensity variance, median pixel intensity and index of dispersion. Derived metrics using a Gaussian image filter (FiltGauss) or an adaptive low-pass Wiener filter (FiltWiener) for image preprocessing.
Laplacian operator[a]<br>LAPM, LAPV	Second derivative based method for passing the high spatial frequencies associated with sharp edges. Applied within user-defined pixel neighborhood (5-by-5 pixel, 15-by-15 pixel). Computing mean and variance.($\in \mathbb{R}$).<br>$L = \frac{\partial^2}{\partial x^2} + \frac{\partial^2}{\partial y^2} = \frac{4}{\alpha+1} \begin{bmatrix} \frac{\alpha}{4} & \frac{1-\alpha}{4} & \frac{\alpha}{4} \\ \frac{1-\alpha}{4} & -1 & \frac{1-\alpha}{4} \\ \frac{\alpha}{4} & \frac{1-\alpha}{4} & \frac{\alpha}{4} \end{bmatrix}$

Diagonal Laplacian[b] LAPD	Second derivative based method for passing the high spatial frequencies associated with sharp edges. LAPD $= \frac{1}{NM}\sum^{M}\sum^{N}\lvert F(M_1)\rvert + \lvert F(M_2)\rvert + \lvert F(M_3)\rvert + \lvert F(M_1^T)\rvert$ $M_1 = [-1, 2, -1]$ $M_2 = \frac{1}{\sqrt{2}}\begin{bmatrix} 0 & 0 & -1 \\ 0 & 2 & 0 \\ -1 & 0 & 0 \end{bmatrix}$, $M_3 = \frac{1}{\sqrt{2}}\begin{bmatrix} -1 & 0 & 0 \\ 0 & 2 & 0 \\ 0 & 0 & -1 \end{bmatrix}$
Otsu Treshold[a] [9] OtsuT, OtsuEM	Threshold (OtsuT) that minimizes the intraclass variance calculated through the weighted sum of variances of the two classes (background and foreground)and effectiveness metric (OtsuEM).
Prewitt operator[a] [10] PRWI, PRWM, PRWV	Measurement based on edge intensities from using a gradient-based method assessing discrete differences in the first derivative. Applied operator in horizontal (G_n) and vertical (G_m) direction (3-by-3 pixel). $G_n = \begin{bmatrix} 1 & 0 & -1 \\ 1 & 0 & -1 \\ 1 & 0 & -1 \end{bmatrix} * I$, $G_m = \begin{bmatrix} 1 & 1 & 1 \\ 0 & 0 & 0 \\ -1 & -1 & -1 \end{bmatrix} * I$

Local range of image[a] RngM, RngV	Transformation where each output pixel ($I_{\mathrm{R}}(m,n)$). contains the range value (maximum value − minimum value) of a user-defined pixel neighborhood of k pixels (7-by-7, 39-by-39, 71-by-71). Derived metrics using a Gaussian image filter (RngGauss) for image pre-processing. $\mathrm{RngM} = \frac{1}{NM}\sum^{M}\sum^{N} I_R$, $\mathrm{RngV} = \frac{1}{NM-1}\sum^{M}\sum^{N} \lvert I_R - \bar{I_R}\rvert^2$ $I_R(m,n) = \max(I(k)) - \min(I(k))$
Spatial frequency[b] [11] SFRQ	Measurement of X gray level intensity of its pixel neighborhood (5-by-5 pixel, 15-by-15 pixel). ($\in \mathbb{R}$). $\mathrm{SFRQ} = \sqrt{I_x^2 + I_y^2}$ $I_y = \sqrt{\frac{1}{MN}\sum_{m=0}^{M-1}\sum_{n=1}^{N-1}[I(m,n) - I(m,n-1)]^2}$ $I_x = \sqrt{\frac{1}{MN}\sum_{n=0}^{N-1}\sum_{m=1}^{M-1}[I(m,n) - I(m-1,n)]^2}$
Sobel operator[a] [12] SOBL	Measurement based on edge intensities from using a gradient-based method assessing discrete differences in the first derivative. Applied operator in horizontal (G_x) and vertical (G_y) direction (3-by-3 pixel). ($\in \mathbb{R}$). $G_x = \begin{bmatrix} 1 & 0 & -1 \\ 2 & 0 & -2 \\ 1 & 0 & -1 \end{bmatrix} * I$, $G_y = \begin{bmatrix} 1 & 2 & 1 \\ 0 & 0 & 0 \\ -1 & -2 & -1 \end{bmatrix} * I$

Tenengrad[b] [4]<br>TENG, TENV	Method to estimate the gradient magnitude for each pixel (S) and to calculate the sum of all magnitudes greater than threshold (T). $G_x(m,n)$ and $G_y(m,n)$ are the convolution of the input image $I(m,n)$ with the Sobel operator (see SOBL).<br>$\text{TENG} = \frac{1}{NM}\sum^{M}\sum^{N}[S(m,n)]^2$ for $S(m,n) > T$<br>$S(m,n) = \sqrt{[G_x(m,n)]^2 + [G_y(m,n)]^2}$
Vollath's correlation[b] [2]<br>VOLA	Measurement derived from the autocorrelation function.<br>$\text{VOLA} = \sum_{m=1}^{M-1}\sum_{n=1}^{N} I(m,n) \cdot I(m+1,n) - \sum_{m=1}^{M-2}\sum_{n=1}^{N} I(m,n) \cdot I(m+2,n)$
2D Wavelet decomposition[a] [13]<br>WAVS, WAVV, WAVR	Measurement based on a 3 level 2D wavelet decomposition using Daubechies D6 wavelet filter, with high-pass and low-pass filtering (D diagonal, V vertical and H horizontal and A_1- A_3 approximation coefficient level 1-3).<br>$\text{WAVS} = \frac{1}{NM}\sum^{M}\sum^{N}\lvert D(m,n)\rvert + \lvert L(m,n)\rvert + \lvert H(m,n)\rvert$<br>$\text{WAVV} = \frac{1}{NM}\sum^{M}\sum^{N}\lvert D(m,n) - \bar{D}\rvert + \lvert L(m,n) - \bar{L}\rvert + \lvert H(m,n) - \bar{H}\rvert$<br>$\text{WAVR} = W_H/W_L$, $W_H = \frac{1}{NM}\sum^{M}\sum^{N}[H^2 + V^2 + D^2]$, $W_L = \frac{1}{NM}\sum^{M}\sum^{N}[A_1 + A_2 + A_3]$

S1.3. Image feature resolution

Table S3: Ranking of 80 extracted images features according to their minimum peak resolution (R_{min}) with no frame averaging (k_{av} = 1) comparing the data resolution between 8 discrete changes in crystal suspension density between x_c = 37.8 mg/mL and 0.0 mg/mL (S = 0.9). The Pearson correlation coefficient (PCC) quantifies the linear correlation between each image feature and the crystal suspension density (x_c) as the response variable.

**Feature**	**Rank**	$\mathbf{R}_{mean}$	$\mathbf{R}_{std}$	$\mathbf{R}_{min}$	$\mathbf{R}_{max}$	**PCC**
WAVR	1	1.54	0.47	1.20	2.63	0.99
SFRQ_015	2	1.36	0.50	0.77	2.33	0.47
SFRQ_050	3	1.35	0.49	0.77	2.31	0.47
WAVS	4	1.22	0.50	0.60	1.96	0.38
FiltGaussE	5	1.55	1.58	0.55	5.39	-0.97
HISE	6	1.61	1.47	0.49	5.11	-0.98
FiltWienerE	7	1.17	1.13	0.48	3.91	-0.98
LAPM_015	8	1.20	0.53	0.48	1.97	0.38
Hist90	9	1.00	0.54	0.37	1.59	-0.89
HistSumQ1	10	1.18	0.53	0.37	1.81	0.63
Hist75	11	1.12	0.50	0.34	1.80	-0.88
EntFiltM_071	12	1.78	2.02	0.34	6.64	-0.98
GLVA	13	1.08	0.61	0.30	2.13	-0.87
IntS	14	1.08	0.61	0.30	2.13	-0.87
OtsuT	15	1.18	0.66	0.30	2.10	-0.91
IntM	16	1.24	0.58	0.30	2.06	-0.91
GLCM_ENGM	17	1.48	2.10	0.29	6.50	0.93
RngGaussM_071	18	1.06	0.68	0.29	2.51	-0.90

IntV	19	0.91	0.49	0.28	1.46	-0.77
FiltWienerM	20	1.07	0.56	0.27	1.68	-0.87
FiltGaussM	21	1.10	0.54	0.27	1.70	0.32
WAVV	22	1.06	0.51	0.26	1.72	0.14
RngGaussM_157	23	0.99	0.69	0.26	2.54	-0.91
Hist50	24	1.28	0.58	0.25	1.97	-0.89
IntMd	25	1.32	0.60	0.25	1.96	-0.89
LAPM_023	26	1.93	2.35	0.24	7.65	-0.97
RngM_071	27	1.09	0.68	0.24	2.38	-0.91
GRAS	28	1.17	0.47	0.23	1.71	0.15
GRAE	29	1.15	0.45	0.22	1.69	0.17
RngM_039	30	1.13	0.69	0.20	2.27	-0.90
GDER	31	0.79	0.42	0.20	1.33	-0.76
FiltGaussV	32	1.03	0.47	0.20	1.63	0.13
HELM_005	33	1.21	0.56	0.19	1.89	0.84
HistSpan	34	1.40	1.06	0.19	3.38	0.47
RngGaussV_071	35	0.45	0.33	0.18	1.18	-0.72
RngE_007	36	1.21	1.51	0.17	4.82	-0.94
RngV_007	37	0.53	0.30	0.16	1.03	-0.74
EntFiltV_007	38	0.51	0.36	0.15	1.17	-0.62
GLCM_CONTM	39	0.67	0.46	0.14	1.36	-0.86
LAPV	40	2.52	4.58	0.14	13.80	-0.95
HELM_015	41	1.12	0.65	0.12	1.88	0.84
PRWI	42	0.94	0.57	0.12	1.62	-0.76
SOBL	43	0.96	0.58	0.12	1.64	-0.78
GLCM_HOMM	44	0.90	0.87	0.11	2.58	0.89
PRWV	45	0.94	0.60	0.11	1.69	-0.74

FiltWienerV	46	0.88	0.49	0.11	1.40	-0.75
TENG	47	0.94	0.60	0.11	1.70	-0.74
GLLV_005	48	0.64	0.57	0.11	1.49	-0.55
PRWM	49	0.71	0.65	0.10	1.66	-0.53
TENV	50	0.71	0.64	0.10	1.66	-0.53
Hist25	51	1.54	1.15	0.09	3.99	-0.91
GLCM_HOMV	52	0.23	0.13	0.09	0.46	-0.54
GLCM_ENGV	53	0.17	0.08	0.09	0.30	-0.48
RngE_039	54	1.18	1.97	0.09	5.96	-0.95
HISR	55	0.56	0.49	0.09	1.57	-0.90
GRAT	56	1.00	0.54	0.08	1.68	0.00
GLCM_CONTV	57	0.18	0.07	0.08	0.32	-0.40
GLLV_015	58	0.43	0.28	0.08	0.77	-0.59
IntIdxD	59	0.98	0.78	0.07	2.19	-0.80
BREN	60	0.94	0.61	0.07	1.70	-0.67
RngGaussV_157	61	0.26	0.15	0.06	0.46	-0.59
RngM_007	62	1.10	0.67	0.06	1.79	-0.78
RngGaussE_157	63	0.39	0.30	0.05	0.89	-0.92
EntFiltM_007	64	1.31	0.88	0.04	3.05	-0.62
Hist10	65	1.97	2.33	0.04	7.52	-0.92
GLCM_CORRV	66	0.07	0.03	0.03	0.13	-0.40
GLCM_CORRM	67	0.62	0.48	0.03	1.51	-0.94
RngV_071	68	0.32	0.34	0.02	1.09	-0.74
EntFiltV_071	69	0.33	0.22	0.02	0.73	-0.64
RngV_039	70	0.39	0.46	0.02	1.46	-0.79
RngE_071	71	0.70	0.94	0.01	2.82	-0.93
OtsuEM	72	0.70	0.66	0.00	1.61	0.05

RngGaussE_071	73	0.90	0.96	0.00	3.12	-0.96
VOLA	74	0.30	0.34	0.00	1.05	-0.73
EntFiltE_007	75	0.09	0.11	0.00	0.30	-0.37
EntFiltE_071	76	0.03	0.04	0.00	0.11	-0.13
HistSumQ4	77	0.50	1.13	0.00	3.27	-0.51
HistSumQ3	78	0.29	0.36	0.00	0.94	-0.73
HistSumQ2	79	0.46	0.76	0.00	2.23	-0.86
HistSumBK	80	0.98	0.78	0.00	1.89	0.80

Table S4: Ranking of 80 extracted images features according to their minimum peak resolution (R_{min}) with a frame averaging of k_{av} = 5 comparing the data resolution between 8 discrete changes in crystal suspension density between x_c = 37.80 mg/mL and 0 mg/mL (S = 0.9). The Pearson correlation coefficient (PCC) quantifies the linear correlation between each image feature and the crystal suspension density (x_c) as the response variable.

**Feature**	**Rank**	$\mathbf{R}_{mean}$	$\mathbf{R}_{std}$	$\mathbf{R}_{min}$	$\mathbf{R}_{max}$	**PCC**
WAVR	1	2.68	1.01	1.91	5.01	0.99
WAVS	2	1.93	0.87	1.17	3.61	0.39
FiltGaussE	3	3.03	3.27	1.13	11.02	-0.98
SFRQ_015	4	2.13	0.98	1.03	4.11	0.48
SFRQ_050	5	2.12	0.97	1.03	4.07	0.48
LAPM_015	6	1.88	0.91	1.02	3.63	0.39
FiltWienerE	7	2.49	2.62	1.00	8.84	-0.98
HISE	8	2.92	3.00	1.00	10.21	-0.99

Hist90	9	1.72	0.74	0.79	2.69	-0.93
HistSumQ1	10	1.98	0.81	0.74	2.97	0.66
GLVA	11	2.09	1.39	0.71	5.21	-0.92
IntS	12	2.09	1.39	0.71	5.21	-0.92
Hist75	13	1.91	0.69	0.70	2.99	-0.92
EntFiltM_071	14	3.22	4.20	0.70	13.48	-0.99
RngGaussM_071	15	2.14	1.65	0.67	5.97	-0.94
OtsuT	16	2.40	1.46	0.67	4.67	-0.95
IntV	17	1.73	0.88	0.66	3.20	-0.86
GLCM_ENGM	18	2.53	3.29	0.63	10.39	0.93
IntM	19	2.16	1.10	0.61	4.28	-0.94
FiltGaussM	20	1.71	0.87	0.60	3.02	0.34
FiltWienerM	21	1.87	1.13	0.58	4.11	-0.94
RngGaussM_157	22	2.07	1.64	0.58	5.87	-0.94
WAVV	23	1.67	0.79	0.55	2.76	0.15
RngM_071	24	2.12	1.59	0.50	5.62	-0.95
GRAS	25	1.89	0.84	0.49	3.00	0.16
Hist50	26	2.16	1.03	0.49	3.92	-0.92
IntMd	27	2.30	1.07	0.49	3.92	-0.93
GRAE	28	1.86	0.79	0.47	2.71	0.18
GDER	29	1.54	0.78	0.46	2.88	-0.87
LAPM_023	30	3.36	4.28	0.45	13.83	-0.98
RngM_039	31	2.10	1.56	0.43	5.45	-0.96
FiltGaussV	32	1.63	0.69	0.41	2.55	0.14
RngGaussV_071	33	0.99	0.73	0.41	2.59	-0.84
RngE_007	34	2.61	3.74	0.40	11.75	-0.96
HistSpan	35	2.29	1.77	0.40	5.84	0.49

HELM_005	36	1.99	0.88	0.38	3.13	0.84
RngV_007	37	1.14	0.66	0.37	2.41	-0.86
EntFiltV_007	38	0.98	0.51	0.33	1.73	-0.78
LAPV	39	5.21	10.08	0.30	30.09	-0.95
GLCM_CONTM	40	1.34	1.00	0.30	3.25	-0.93
PRWI	41	1.58	0.92	0.27	2.67	-0.90
SOBL	42	1.62	0.98	0.27	2.90	-0.91
FiltWienerV	43	1.55	0.87	0.26	2.81	-0.89
GLCM_HOMM	44	1.79	1.90	0.26	5.92	0.90
PRWV	45	1.55	0.93	0.26	2.71	-0.91
TENG	46	1.54	0.94	0.25	2.72	-0.91
GLLV_005	47	1.15	0.89	0.24	2.53	-0.77
HELM_015	48	1.84	1.03	0.23	3.12	0.84
PRWM	49	1.19	0.93	0.22	2.66	-0.76
TENV	50	1.19	0.92	0.21	2.65	-0.76
RngE_039	51	2.59	4.36	0.21	13.21	-0.96
GLCM_HOMV	52	0.55	0.32	0.20	1.14	-0.78
GLCM_ENGV	53	0.37	0.19	0.19	0.72	-0.72
GLCM_CONTV	54	0.40	0.17	0.18	0.73	-0.65
HISR	55	1.28	1.13	0.18	3.64	-0.93
GRAT	56	1.55	0.77	0.18	2.66	0.01
Hist25	57	2.57	2.14	0.18	7.37	-0.94
GLLV_015	58	0.94	0.59	0.17	1.68	-0.79
IntIdxD	59	1.86	1.61	0.16	5.14	-0.87
BREN	60	1.50	0.96	0.16	2.67	-0.85
RngGaussE_157	61	0.87	0.68	0.13	2.00	-0.95
RngM_007	62	1.87	1.34	0.13	4.35	-0.86

RngGaussV_157	63	0.57	0.34	0.11	1.03	-0.72
EntFiltM_007	64	2.10	1.69	0.08	5.82	-0.64
Hist10	65	3.30	4.14	0.07	13.25	-0.95
GLCM_CORRM	66	1.25	0.84	0.06	2.43	-0.95
GLCM_CORRV	67	0.16	0.08	0.06	0.28	-0.69
EntFiltV_071	68	0.72	0.49	0.04	1.62	-0.83
RngV_039	69	0.91	1.08	0.04	3.41	-0.89
RngV_071	70	0.72	0.75	0.04	2.41	-0.84
RngE_071	71	1.55	2.13	0.02	6.39	-0.94
OtsuEM	72	1.22	1.05	0.01	2.91	0.05
RngGaussE_071	73	1.87	1.98	0.00	6.42	-0.96
VOLA	74	0.70	0.81	0.00	2.48	-0.86
EntFiltE_007	75	0.21	0.26	0.00	0.69	-0.64
EntFiltE_071	76	0.06	0.09	0.00	0.25	-0.28
HistSumQ4	77	1.07	2.36	0.00	6.89	-0.71
HistSumQ3	78	0.55	0.63	0.00	1.56	-0.77
HistSumQ2	79	1.01	1.76	0.00	5.20	-0.91
HistSumBK	80	1.53	1.22	0.00	3.02	0.80

S1.4. Application (A1) - nucleation detection

Table S5: Ranking of all 80 extracted image features for the detection of *clear* (complete dissolution) and *cloud* points (initial nucleation) assessed against a user-defined ground truth for three image datasets. The features were ranked according to their evaluated mean square temperature error (MSE) using a divergence criterion against the median background image feature signal (clear images, $m_{y_i,bk}$) with $y_i > |m_{y_i,bk} \pm n_{\mathrm{Nuc}} \cdot \sigma_{y_i,bk}|$ at $n_{\mathrm{Nuc}} = 4$, 8 and 16. The ranking includes optimised signal processing parameters for a Hampel filter and a moving average filter (Savitzky-Golay, order 1) optimised during a grid-search with k_{Hpl} and $k_{\mathrm{SG}} \in [5, 41]$ with a step-size of 2 and $n_{\mathrm{Hpl},\sigma} \in [0.1, 6]$ with a step-size of 0.4.

**Feature**	**Rank**	**MSE [°C^2]**				**Data Processing**		
		Avg	$\pm 4\sigma$	$\pm 8\sigma$	$\pm 16\sigma$	k_{Hpl}	$n_{\mathrm{Hpl},\sigma}$	k_{SG}
HELM_005	1	0.05	0.05	0.05	0.05	29	0.1	11
BREN	2	0.06	0.06	0.05	0.07	5	3.3	19
PRWV	3	0.06	0.06	0.04	0.09	33	5.3	9
TENG	4	0.06	0.06	0.04	0.09	37	5.3	11
VOLA	5	0.07	0.04	0.03	0.14	19	4.9	15
SOBL	6	0.07	0.06	0.03	0.12	9	5.3	13
RngGaussM_157	7	0.07	0.07	0.04	0.10	7	5.7	17
PRWI	8	0.07	0.05	0.04	0.12	7	5.7	11
RngGaussM_071	9	0.07	0.11	0.05	0.06	5	2.9	17
LAPV	10	0.07	0.09	0.04	0.09	7	0.9	11
RngE_007	11	0.07	0.10	0.04	0.08	9	0.5	7
FiltWienerM	12	0.07	0.06	0.04	0.12	41	3.7	17
EntFiltV_007	13	0.08	0.10	0.04	0.09	15	5.7	13

LAPM_023	14	0.08	0.11	0.06	0.07	19	2.5	9
RngV_007	15	0.08	0.10	0.05	0.09	7	4.9	15
OtsuEM	16	0.08	0.04	0.09	0.13	21	2.9	13
RngM_071	17	0.08	0.10	0.04	0.11	7	4.9	15
HELM_015	18	0.09	0.09	0.08	0.09	11	2.1	5
HistSumQ1	19	0.09	0.09	0.09	0.09	11	0.1	17
RngM_039	20	0.09	0.14	0.05	0.07	15	3.7	15
GDER	21	0.09	0.10	0.07	0.10	11	3.7	7
RngV_039	22	0.10	0.10	0.05	0.14	5	0.1	9
RngM_007	23	0.10	0.08	0.05	0.16	33	1.3	17
RngGaussV_071	24	0.10	0.16	0.05	0.11	7	0.1	11
RngV_071	25	0.12	0.12	0.10	0.14	5	1.3	15
TENV	26	0.13	0.19	0.07	0.12	5	0.1	9
PRWM	27	0.13	0.18	0.09	0.11	5	1.7	17
FiltWienerV	28	0.13	0.11	0.11	0.18	5	0.9	41
RngE_039	29	0.13	0.15	0.11	0.14	21	2.9	13
RngE_071	30	0.14	0.11	0.08	0.23	15	2.9	13
RngGaussE_071	31	0.14	0.10	0.06	0.27	5	0.1	5
GLLV_005	32	0.17	0.24	0.14	0.12	7	0.1	13
HISR	33	0.25	0.20	0.17	0.39	19	1.3	17
RngGaussE_157	34	0.28	0.27	0.17	0.41	11	1.7	29
HistSumQ2	35	0.32	0.42	0.26	0.27	39	5.7	5
GLLV_015	36	0.35	0.41	0.35	0.30	7	4.1	35
GLCM_CORRV	37	0.38	0.29	0.37	0.46	5	0.5	41
RngGaussV_157	38	0.43	0.57	0.39	0.34	29	4.1	19
GLCM_CONTM	39	0.68	0.28	0.55	1.21	33	1.7	41
GLCM_ENGV	40	0.90	0.40	0.77	1.53	7	5.7	41

OtsuT	41	2.13	1.33	2.05	3.00	41	0.5	41
IntMd	42	2.33	0.81	2.50	3.67	13	5.7	41
WAVV	43	3.84	9.53	0.65	1.33	23	3.7	5
EntFiltE_007	44	4.63	4.63	4.63	4.63	11	0.1	41
EntFiltV_071	45	4.83	13.49	0.43	0.59	35	5.3	7
EntFiltM_007	46	5.65	16.46	0.18	0.30	15	0.9	35
GRAE	47	6.04	17.49	0.19	0.45	21	1.7	39
FiltGaussV	48	6.05	17.48	0.20	0.46	11	5.7	35
GRAS	49	6.06	17.47	0.24	0.46	11	4.9	35
WAVR	50	6.42	17.70	0.55	1.00	5	1.7	17
FiltGaussM	51	6.43	17.69	0.60	0.99	9	4.5	35
GRAT	52	6.54	18.23	0.53	0.86	5	5.7	21
IntM	53	7.95	17.50	2.48	3.87	13	4.9	39
Hist50	54	8.00	17.52	2.57	3.92	31	4.5	41
GLCM_CONTV	55	8.34	24.00	0.42	0.60	7	4.5	9
HistSumQ4	56	8.40	18.14	3.00	4.06	13	2.5	39
HISE	57	8.40	23.03	0.78	1.41	5	0.9	5
FiltGaussE	58	8.52	23.47	0.72	1.36	5	0.9	5
FiltWienerE	59	9.64	24.36	1.85	2.71	5	1.7	7
HistSumQ3	60	10.11	18.56	4.03	7.75	9	0.5	41
LAPM_015	61	10.14	18.95	1.80	9.67	11	5.7	7
GLCM_HOMM	62	10.28	0.92	1.73	28.20	41	3.3	41
WAVS	63	11.55	19.37	5.70	9.58	9	5.3	7
Hist75	64	11.59	19.89	6.27	8.60	5	0.9	29
EntFiltM_071	65	11.65	34.64	0.08	0.22	5	1.7	5
Hist90	66	13.58	20.36	6.11	14.26	39	4.1	7
Hist25	67	13.62	18.42	18.40	4.03	9	1.3	41

SFRQ_050	68	14.01	19.92	18.59	3.53	11	5.7	39
SFRQ_015	69	14.02	19.94	18.59	3.52	11	5.7	39
Hist10	70	14.74	18.79	21.08	4.36	9	0.9	5
GLVA	71	16.75	47.76	1.05	1.45	25	3.7	5
IntS	72	16.75	47.76	1.05	1.45	25	3.7	5
IntV	73	16.80	47.76	1.20	1.45	25	3.7	5
GLCM_HOMV	74	18.71	54.30	0.80	1.03	5	5.7	9
GLCM_ENGM	75	19.57	24.93	2.81	30.96	7	2.1	25
IntIdxD	76	26.15	54.58	22.58	1.30	9	5.7	7
HistSpan	77	31.21	68.08	23.12	2.43	7	5.3	5
GLCM_CORRM	78	52.29	25.94	53.09	77.85	5	5.3	39
EntFiltE_071	79	Failed						
HistSumBK	80	Failed						

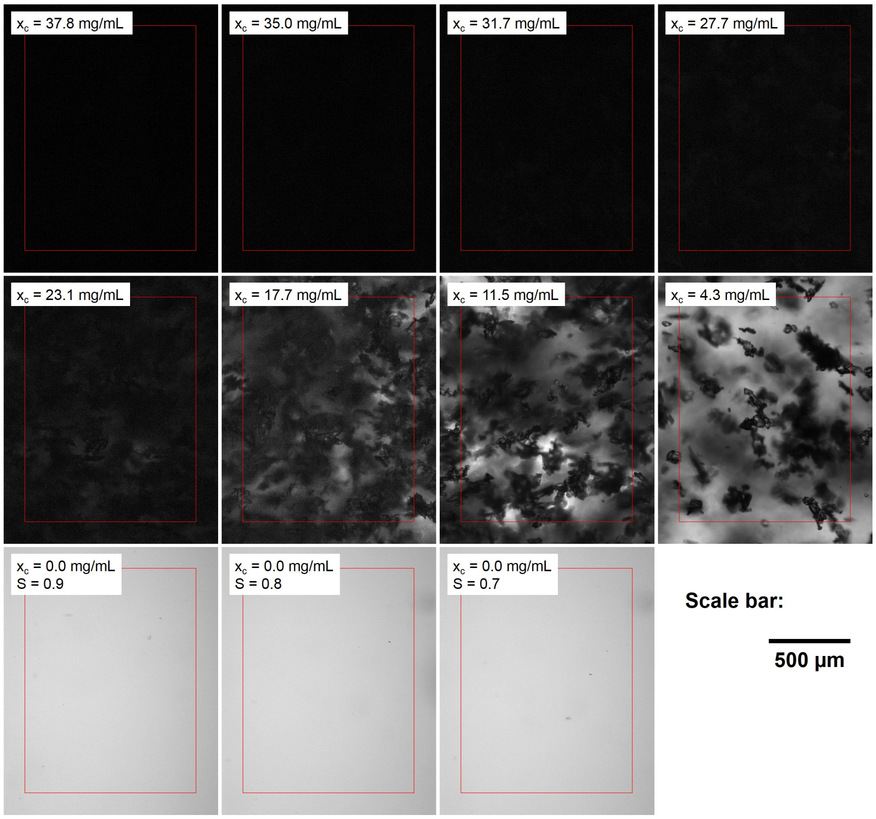

Figure S1: Selected raw images of MFA-DT during stepwise heating at discrete isothermal conditions with changing crystal suspension density (x_c) and supersaturation (S).

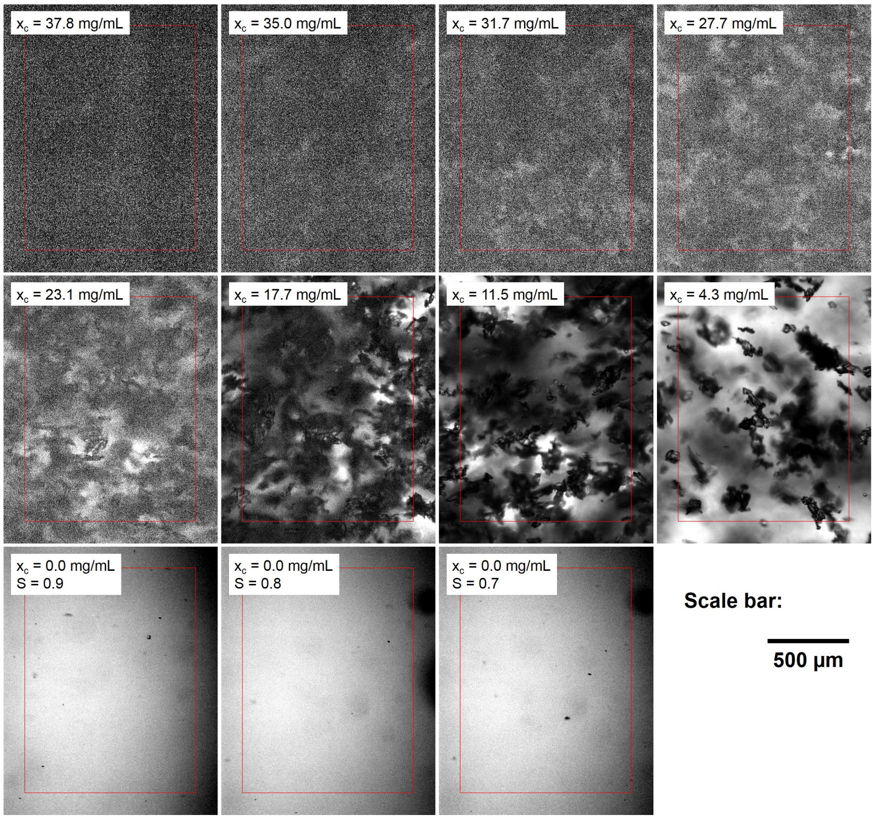

Figure S2: Selected images of MFA-DT during stepwise heating at discrete isothermal conditions with changing crystal suspension density (x_c) and supersaturation (S). The images were further processed (stretched histogram) to visualise for the reader subtle differences in the pixel intensities not directly apparent in the raw images (Fig. S1) for $x_c > 23.1$ mg/mL.

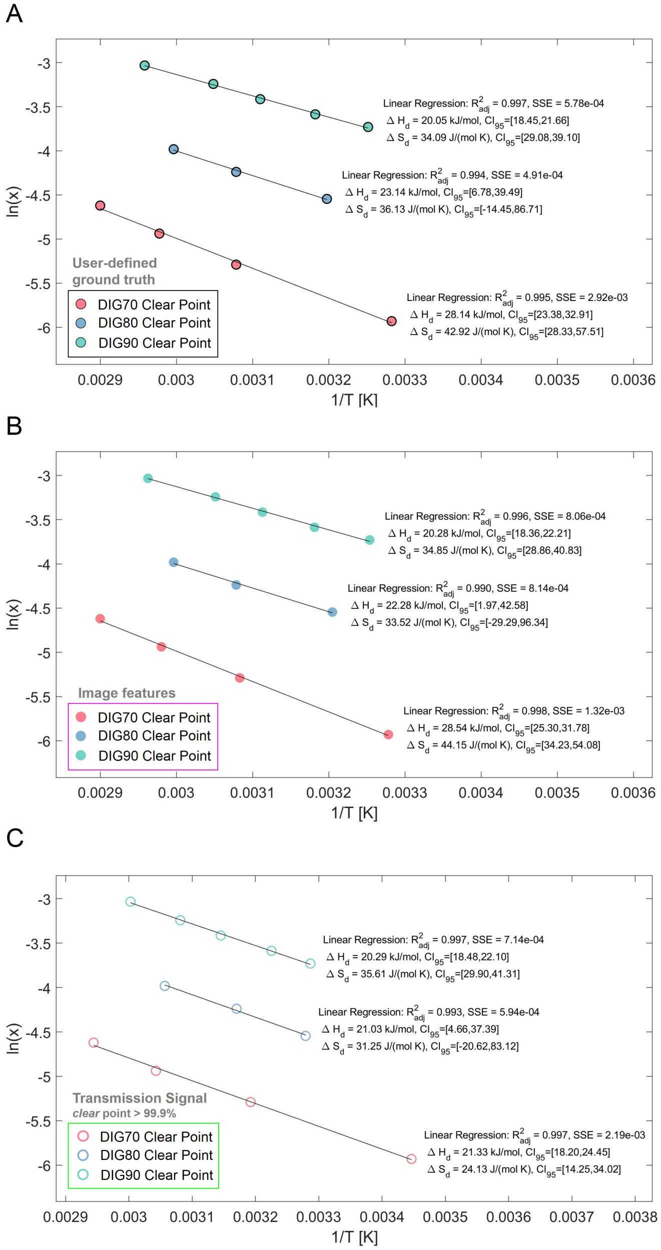

Figure S3: Regression analysis using the linear form expression of the the Van't Hoff solubility equation ($\ln(x) = -\Delta H_\mathrm{d}/R \cdot 1/T + \Delta S_\mathrm{d}/R$) and collected *clear* point data (complete dissolution) from (A) the user-defined ground truth (visual image inspection), (B) image feature-based detection and (C) transmission-based detection.

S1.5. Application (A2) - suspension density prediction

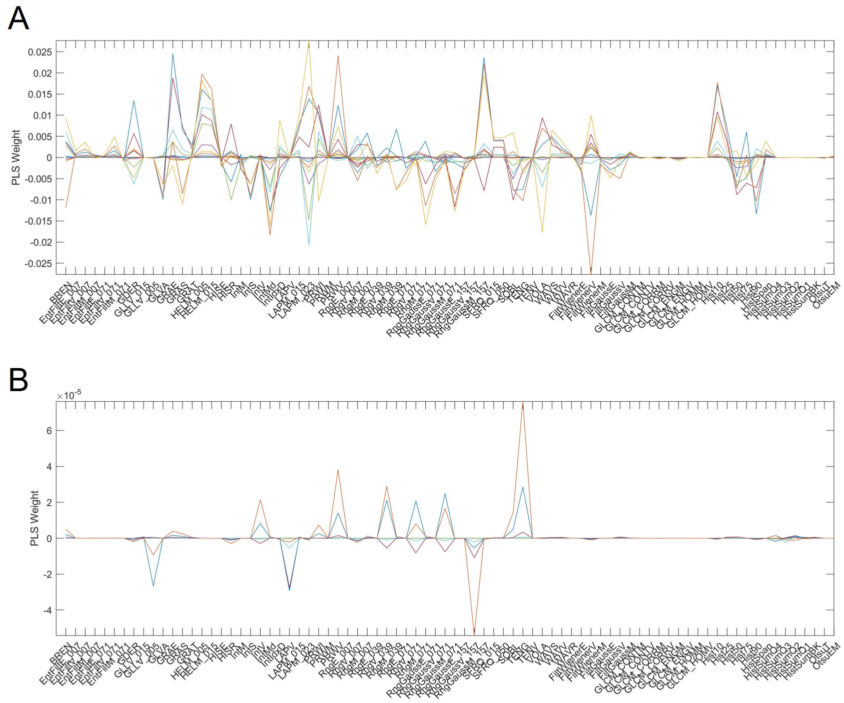

Figure S4: PLSR weights across 80 image features. (A) Using a single dataset MFA-DT for PLSR training/testing ($k_{av} = 5$) with 24 latent variables (ncomp). (B) Using all datasets MFA-70-1 - MFA-90-5 for training and testing ($k_{av} = 5$) with 9 latent variables (ncomp).

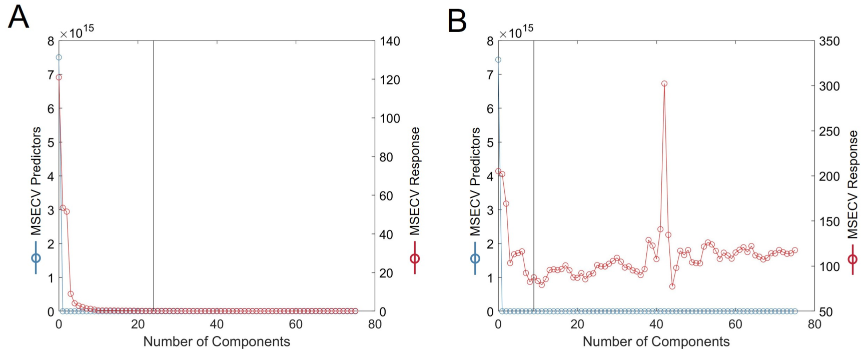

Figure S5: MSECV curves used to optimise number of latent variables (ncomp) for PLSR. (A) Using a single dataset MFA-DT for PLSR training/testing with 10-fold cross-validation ($k_{av} = 5$, ncomp = 24). (B) Using all datasets MFA-70-1 to MFA-90-5 for training with 4-fold cross-validation ($k_{av} = 5$, ncomp = 9) only excluding test datasets MFA-80-3 & MFA-90-5.

References

[1] John F Brenner, Brock S Dew, J Brian Horton, Thomas King, Peter W Neurath, and William D Selles. An automated microscope for cytologic research a preliminary evaluation. *Journal of Histochemistry & Cytochemistry*, 24(1):100–111, 1976.

[2] A. Santos, C. Ortiz De Solórzano, J. J. Vaquero, J. M. Peña, N. Malpica, and F. Del Pozo. Evaluation of autofocus functions in molecular cytogenetic analysis. *Journal of Microscopy*, 188(3):264–272, 1997. ISSN 00222720. doi: 10.1046/j.1365-2818.1997.2630819.x.

[3] Jan Mark Geusebroek, Frans Cornelissen, Arnold W.M. Smeulders, and Hugo Geerts. Robust autofocusing in microscopy. *Cytometry*, 39(1):1–9, 2000. ISSN 01964763. doi: 10.1002/(SICI)1097-0320(20000101)39:1<1::AID-CYTO2>3.0.CO;2-J.

[4] J. L. Pech-Pacheco, G. Cristöbal, J. Chamorro-Martínez, and J. Fernândez-Valdivia. Diatom autofocusing in brightfield microscopy: A comparative study. *Proceedings - International Conference on Pattern Recognition*, 15 (3):314–317, 2000. ISSN 10514651. doi: 10.1109/icpr.2000.903548.

[5] E. Krotkov and J.-P. Martin. Range from focus. In *Proceedings. 1986 IEEE International Conference on Robotics and Automation*, volume 3, pages 1093–1098. Institute of Electrical and Electronics Engineers, 1986. doi: 10.1109/ROBOT.1986.1087510.

[6] Murali Subbarao, Tae-Sun Choi, and Arman Nikzad. Focusing techniques. In Bruce G. Batchelor, Susan Snell Solomon, and Frederick M. Waltz, editors, *Machine Vision Applications, Architectures, and Systems Integration*, volume 1823, pages 163–174, nov 1992. doi: 10.1117/12.132073.

[7] Franz Stephan Helmli and Stefan Scherer. Adaptive shape from focus with an error estimation in light microscopy. In *ISPA 2001. Proceedings of the 2nd International Symposium on Image and Signal Processing and Analysis*, pages 188–193. IEEE, 2001.

[8] Lawrence Firestone, Kitty Cook, Kevin Culp, Neil Talsania, and Kendall Preston. Comparison of autofocus methods for automated microscopy. *Cytometry*, 12(3):195–206, 1991. ISSN 10970320. doi: 10.1002/cyto.990120302.

[9] N Otsu. A Threshold Selection Method from Gray-Level Histograms. *IEEE Transactions on Systems, Man and Cybernetics*, 20(1):62–66, 1975.

[10] Judith M S Prewitt. *Object enhancement and extraction*, volume 10. Academic Press New York, 1970.

[11] Ahmet M. Eskicioglu and Paul S. Fisher. Image Quality Measures and

Their Performance. *IEEE Transactions on Communications*, 43(12):2959–2965, 1995. ISSN 00906778. doi: 10.1109/26.477498.

[12] M. R. B. Clarke, Richard O. Duda, and Peter E. Hart. Pattern Classification and Scene Analysis. *Journal of the Royal Statistical Society. Series A (General)*, 137(3):442, 1974. ISSN 00359238. doi: 10.2307/2344977.

[13] Yves Meyer. *Wavelets and Operators: Volume 1*. Number 37. Cambridge university press, 1992.
